# A manufacturable platform for photonic quantum computing

**DOI:** 10.1038/s41586-025-08820-7

**Published:** 2025-02-26

**Authors:** PsiQuantum team, Koen Alexander, Avishai Benyamini, Dylan Black, Damien Bonneau, Stanley Burgos, Ben Burridge, Hugo Cable, Geoff Campbell, Gabriel Catalano, Alejandro Ceballos, Chia-Ming Chang, Sourav Sen Choudhury, C. J. Chung, Fariba Danesh, Tom Dauer, Michael Davis, Eric Dudley, Ping Er-Xuan, Josep Fargas, Alessandro Farsi, Colleen Fenrich, Jonathan Frazer, Masaya Fukami, Yogeeswaran Ganesan, Gary Gibson, Mercedes Gimeno-Segovia, Sebastian Goeldi, Patrick Goley, Ryan Haislmaier, Sami Halimi, Paul Hansen, Sam Hardy, Jason Horng, Matthew House, Hong Hu, Mehdi Jadidi, Vijay Jain, Henrik Johansson, Thomas Jones, Vimal Kamineni, Nicholas Kelez, Ravi Koustuban, George Kovall, Peter Krogen, Nikhil Kumar, Yong Liang, Nicholas LiCausi, Dan Llewellyn, Kimberly Lokovic, Michael Lovelady, Vitor Riseti Manfrinato, Ann Melnichuk, Gabriel Mendoza, Brad Moores, Shaunak Mukherjee, Joseph Munns, Francois-Xavier Musalem, Faraz Najafi, Jeremy L. O’Brien, J. Elliott Ortmann, Sunil Pai, Bryan Park, Hsuan-Tung Peng, Nicholas Penthorn, Brennan Peterson, Gabriel Peterson, Matt Poush, Geoff J. Pryde, Tarun Ramprasad, Gareth Ray, Angelita Viejo Rodriguez, Brian Roxworthy, Terry Rudolph, Dylan J. Saunders, Pete Shadbolt, Deesha Shah, Andrea Bahgat Shehata, Hyungki Shin, Jeffrey Sinsky, Jake Smith, Ben Sohn, Young-Ik Sohn, Gyeongho Son, Mario C. M. M. Souza, Chris Sparrow, Matteo Staffaroni, Camille Stavrakas, Vijay Sukumaran, Davide Tamborini, Mark G. Thompson, Khanh Tran, Mark Triplett, Maryann Tung, Andrzej Veitia, Alexey Vert, Mihai D. Vidrighin, Ilya Vorobeichik, Peter Weigel, Matthew Wingert, Jamie Wooding, Xinran Zhou

**Affiliations:** 1 PsiQuantum Corp., Palo Alto, CA USA; 2 PsiQuantum Ltd., Daresbury, UK

**Keywords:** Qubits, Other photonics, Photonic devices

## Abstract

Although holding great promise for low noise, ease of operation and networking^1^, useful photonic quantum computing has been precluded by the need for beyond-state-of-the-art components, manufactured by the millions^2–6^. Here we introduce a manufacturable platform^7^ for quantum computing with photons. We benchmark a set of monolithically integrated silicon-photonics-based modules to generate, manipulate, network and detect heralded photonic qubits, demonstrating dual-rail photonic qubits with 99.98% ± 0.01% state preparation and measurement fidelity, Hong–Ou–Mandel (HOM) quantum interference between independent photon sources with 99.50% ± 0.25% visibility, two-qubit fusion with 99.22% ± 0.12% fidelity and a chip-to-chip qubit interconnect with 99.72% ± 0.04% fidelity, conditional on photon detection and not accounting for loss. We preview a selection of next-generation technologies: low-loss silicon nitride (SiN) waveguides and components to address loss, as well as fabrication-tolerant photon sources, high-efficiency photon-number-resolving detectors (PNRDs), low-loss chip-to-fibre coupling and barium titanate (BTO) electro-optic phase shifters for high-performance fast switching.

## Abstract (web summary)

A manufacturable platform for quantum computing with photons is introduced and a set of monolithically integrated silicon-photonics-based modules is benchmarked, demonstrating dual-rail photonic qubits with performance close to thresholds required for operation.

## Main

It has long been understood that useful quantum computers will require error correction for fault-tolerant operation and, therefore, on the order of millions of physical qubits^8^. Owing to their intrinsic low-noise properties, photons have been used to implement many of the foundational demonstrations of superposition, entanglement, logic gates, algorithms and so on^1^. However, large-scale photonic quantum computing has so far been precluded by several outstanding and challenging requirements.

Since the earliest proposals for fault-tolerant optical quantum computers^2–6^, it has been clear that a very large number of photonic components would be required for any useful system^9,10^. Furthermore, to satisfy the requirements of error-correcting codes, these components should also perform beyond the state of the art of conventional integrated photonics^9,10^ and must also extend outside the scope of a typical photonics library, introducing non-standard devices—most notably high-efficiency single-photon detectors^11,12^. The need for a very large number of near-identical devices motivates an emphasis on fabrication using conventional, high-volume semiconductor manufacturing processes^7^. Finally, these devices must be integrated in an extensive system—demanding fast control electronics, high-power cryogenic cooling to support the operation of superconducting detectors and low-loss, high-fidelity networking of qubits between modules.

In this paper, we describe a technology stack and basic building blocks for photonic quantum computing, demonstrating the crucial functionalities of qubit generation, manipulation, detection and networking, including single-photon sources, waveguide-integrated superconducting single-photon detectors, single-qubit state preparation and measurement (SPAM), chip-to-chip qubit interconnects, two-photon quantum interference and two-qubit fusion, all at telecommunications (C band) wavelengths. These constitute the basic operations required for most approaches to photonic quantum computing^2–6,9^, including fusion-based quantum computing (FBQC; recently introduced in ref. ^13^). These components are fabricated in a commercial semiconductor foundry^14^, using a fully integrated 300-mm silicon photonics process flow, with all operations on-chip.

To enter the fault-tolerant regime of operation will require a technology stack with improved component performance and further functionality. To this end, we have developed and present next-generation components, with SiN waveguide losses as low as 0.5 ± 0.3 dB m^−1^, splitters and crossings with 0.5 ± 0.2 mdB and 1.2 ± 0.4 mdB loss, respectively, and fibre-to-chip coupling losses as low as 52 ± 12 mdB. The quantum benchmarking results presented here are conditional on photon detection and the photon production is heralded but non-deterministic. To overcome non-determinism in photonic quantum computation, a fast optical switch for multiplexing is required^15–18^. Here we introduce BTO switches into our technology for this purpose, with a loss-voltage product of 0.33 ± 0.02 dB.V. Furthermore, we demonstrate robust photon sources capable of indistinguishable photon generation over a ±400-pm resonance shift and waveguide-integrated photon-number-resolution detectors with 98.9% single-photon median efficiency and up to four-photon resolution. When taken together, these new and improved components constitute a feature-complete set of photonic building blocks having all of the functionality necessary to enable future fault-tolerant photonic quantum computing systems.

## Technology stack and building blocks

**Fig. 1:**
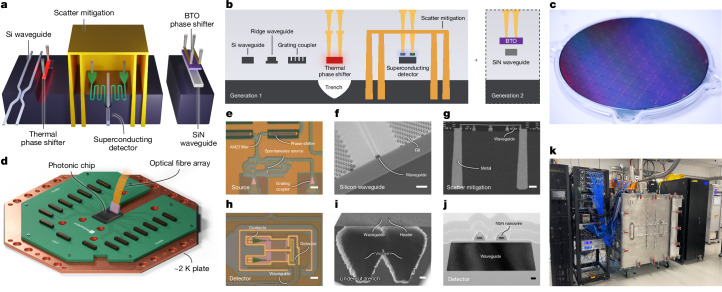
Manufacturable integrated quantum photonic stack. **a**,**b**, Schematics of key components and process modules. We highlight (on the right) further process steps included in our next-generation platform. **c**, A 300-mm wafer containing single-photon sources, superconducting single-photon detectors and quantum benchmarking circuits. **d**, A cryogenic assembly containing a photonic die, heat spreader, electronic PCB and 100-channel telecommunications fibre attach unit. **e**–**j**, Optical micrograph, scanning electron microscope or transmission electron microscopy images of: photon source (top-down) (**e**); optical waveguide (cross-section) (**f**); deep/shallow trench scattered light shield (cross-section) (**g**); single-photon detector (top-down) (**h**); thermal isolation trench (cross-section) (**i**); single-photon detector on waveguide (cross-section) (**j**). **k**, Custom cryostat used in benchmarking experiments with >10 W cooling power at 2.2 K. Scale bars, 20 μm (**e**,**h**), 1 μm (**f**,**i**), 10 μm (**g**), 40 nm (**j**). AMZI, asymmetric Mach–Zehnder interferometer.

Silicon photonics is a mature manufacturing technology, built on decades of industrial development for established applications in the communications, medical and automotive sectors^19,20^. We modified an established silicon photonics manufacturing flow to include high-performance single-photon detection and photon-pair generation (Fig. 1). To our knowledge, this is the first realization of an integrated photonic technology platform capable of on-chip generation, manipulation and detection of photonic qubits.

Our baseline quantum photonic technology stack was developed in partnership with GlobalFoundries and is fabricated in their 300-mm, state-of-the-art, high-volume semiconductor foundry. By making use of industrial unit process steps from semiconductor manufacturing in combination with foundry design services, such as optical proximity correction and optimized process design rules, the technology inherits the scalability and performance of a high-volume commercial environment. The manufacturing flow includes more than 20 photolithography levels and hundreds of processing and in-line measurement steps. Critical process modules developed include passive silicon-on-insulator photonic waveguides, a niobium nitride (NbN) superconducting layer for single-photon detection, deep metal-filled trenches for optical noise reduction, resistive heaters for phase control and optical circuit reconfigurability, grating couplers for optical input/output, back-end-of-line copper electrical interconnects and aluminium redistribution layers.

**Fig. 2:**
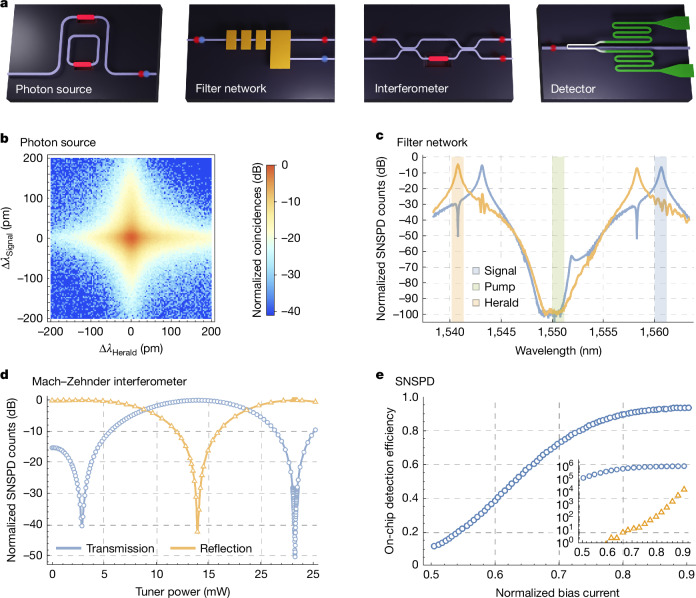
Key building blocks of the platform. **a**, Schematics of photon source, filter network, interferometer and detector. **b**, Measured joint spectral intensity of an interferometrically coupled resonator photon source, indicating a spectral purity of 99.5% (Supplementary Information). **c**, Response of our pump filter network. We shade the pump, signal and herald frequency bands and show the measured herald (orange) and signal (blue) filter spectrum, characterized with on-chip SNSPDs. **d**, Measured response of a Mach–Zehnder interferometer to heralded single-photon illumination on a fully integrated platform. The extinction ratio at the transmission port is >50 dB. The asymmetry in the Mach–Zehnder interferometer response is an artefact of a non-constant step size, which is finer around one feature only. There is no marked variation in performance across a circuit or among different circuits. **e**, Measured on-chip detection efficiency as a function of detector bias current (*I*_B_) normalized by the detector switching current (*I*_SW_) and the detector count rate (blue) and dark count rate (orange) per second (inset) (Supplementary Information).

Using this stack, we build quantum photonic integrated circuits using standard silicon photonic waveguide components, including directional couplers, crossings and thermal phase shifters. We combine these components to produce key building blocks: high-fidelity spontaneous photon-pair sources; interferometers for circuit reconfigurability, qubit manipulation and filtering; and waveguide-integrated single-photon detectors (Fig. 2a). We now outline the performance of each of these building blocks.

### Photon sources

To construct entangled resource states and, in turn, an error-correcting code, photonic quantum computers consume many single photons, which must be generated with high efficiency, well-defined timing and a high repetition rate, whilst also being spectrally pure and indistinguishable^21^. Our single-photon sources use spontaneous four-wave mixing (SFWM)^22^ driven by a pulsed laser pump, for which the generation of a single photon is probabilistic but heralded by the detection of its pair—making a heralded single-photon source (HSPS).

The visibility of two-photon quantum interference, a key operation in photonic quantum computation, is limited by the spectral purity of the heralding single photons, which is determined by the joint spectral intensity of the photon pairs. We use resonator-based optical waveguide structures to tailor the spectral properties of the photon sources to achieve high spectral purity. The pump is aligned to a resonance frequency and single photons are generated at resonant frequencies spaced symmetrically around it, as illustrated with shaded bands in Fig. 2c. Single-ring resonator sources are intrinsically limited to a heralded photon purity of about 93% (ref. ^23^). We circumvent the spectral purity limitation of single resonator sources using interferometrically coupled resonator designs^23^, which we characterized to have a measured spectral purity of 99.5% ± 0.1% without spectral filtering (Fig. 2b and Supplementary Information).

### Photon detection

Photonic quantum computing relies on heralding the creation of quantum states by the detection of correlated photons. Examples include single-photon heralding from pair sources, heralded probabilistic resource state generation and fusion measurements. For fault tolerance, these functions require near-unit-efficiency single-photon detection. We introduced a NbN layer into our photonic stack to enable high-performance manufacturable superconducting nanowire single-photon detectors (SNSPDs)^11,12^.

We use a hairpin-shaped SNSPD design^24^, as depicted in Fig. 2a, with a film thickness of roughly 5 nm, nanowire width of about 90 nm and detector length of ≥80 μm. When operated at approximately 2 K temperature, these detectors exhibit clear plateaus in the photon count rate versus bias current (Fig. 2e and wafer maps in the Supplementary Information), indicating high internal detection efficiency. The on-chip detection efficiency is measured through cryogenic electro-optical measurements of waveguide-integrated SNSPDs (Supplementary Information). Testing of screened SNSPDs yielded a median on-chip efficiency of 93.4% and average value of 88.9% ± 3.5% (Supplementary Information), limited by the hairpin design of the detector.

### Interferometers and filters

Interferometers are a key building block of integrated photonic quantum computing, enabling qubit state preparation and projection, pump filtering, switching networks, resource state generation and fusion measurements. We use combinations of directional couplers, crossings and rings to construct ring resonators and Mach–Zehnder-type interferometers. These components have been optimized through design–test cycles and provide predictable performance guaranteed by strict fabrication process control. An example high-contrast Mach–Zehnder interference fringe, measured with co-integrated HSPS and SNSPDs, is shown in Fig. 2d, with a >50 dB extinction ratio.

Such passive circuits are reconfigurable at low frequencies using thermal phase shifters, which are commonplace in silicon photonics. Although the circuit itself is cooled to cryogenic temperature (to support the operation of integrated superconducting single-photon detectors), the thermal phase shifters reach local temperatures well in excess of 100 °C during operation. Given the available cooling power per unit area at about 2 K cryogenic temperatures, thermal insulation of the phase shifter—using undercut regions etched from the silicon substrate (Fig. 1a,b,i)—is critical to achieve sufficient efficiency. Most if not all of these heaters are made redundant and will ultimately be removed thanks to the second-generation technologies described later in this manuscript—in particular, the fabrication-tolerant source, which will not require tuning, as well as the electro-optic phase shifter, which can operate at GHz rates.

## Integrated heralded single-photon generation and quantum benchmarking circuits

So far, photonic quantum computing platforms have depended on off-chip single-photon sources, off-chip single-photon detectors or both. Although sufficient for demonstration purposes, it is very challenging to achieve the heralding efficiency and component density required for practical fault-tolerant quantum computing without co-integration of the source, filter and heralding detector. Through integration of our key building blocks into our semiconductor platform, we have developed the world’s first fully integrated HSPS—including source, filtering and heralding on the same chip. Using this, we construct benchmarking quantum circuits to quantify single-qubit, two-qubit and chip-to-chip qubit interconnect performance, which is summarized in Table 1.

**Table 1:** Single-qubit and two-qubit performance metrics. Not accounting for loss

	Metric	Experiment value (%)
Single-qubit	SPAM fidelity	99.98 ± 0.01 99.996 ± 0.003*
	Chip-to-chip fidelity	99.72 ± 0.04
Two-qubit	Quantum interference visibility	99.50 ± 0.25
	Bell fidelity	99.22 ± 0.12

*Second SPAM fidelity listed above is measured with bright light and off-chip detectors; see main text.

We selected photonic dies from 300-mm wafers using high-volume in-line and end-of-line electric, optical and electro-optical room-temperature testing, as well as cryogenic electro-optic testing for select parts. For our most complex systems, we package these dies into assemblies (Fig. 1d) together with thermal heat sinks, more than 1,000 electrical connections and up to 200 optical input/output. We house these packages in cryostats with approximately 2 K base temperature and up to 20 W cooling capacity (Fig. 1k).

### HSPS

A high-performance HSPS requires engineered SFWM sources, heralding detectors, as well as a high-performance filter network on-chip, which we now describe. To separate the bright laser pump from the single photons, we require about 100 dB suppression of the pump photons. To achieve this in an integrated circuit, we combine both interferometric in-guide filtering and shielding of the detectors from out-of-guide scattered pump light. In-guide filtering uses a series of first-order and third-order asymmetric Mach–Zehnder interferometers combined with add-drop resonators to select single-source resonances for the herald and the signal photons. Optimizing the free spectral range and coupling values of each element, we achieve pump rejection of 99.1 ± 1.2 dB (Fig. 2c and Supplementary Information) and the simultaneous rejection of unwanted broadband parametric processes. The signal and herald photons are transmitted through filter networks with approximately 1 dB of loss. To suppress scattered light, we locally shield the detectors by encasing them in metal (Fig. 1a,b,g). The shields are constructed from deep and shallow metal-filled trenches and back-end-of-line metals. We observe approximately 115 dB pump power suppression between the pump input and the SNSPDs.

The integrated filters and scattered light shielding, combined with co-integration of SFWM and SNSPDs, allowed for the first demonstration, to our knowledge, of successful on-chip integrated heralded single-photon production, with coincidences-to-accidentals ratios^25^ of up to 3,000 (Supplementary Information).

### SPAM

**Fig. 3:**
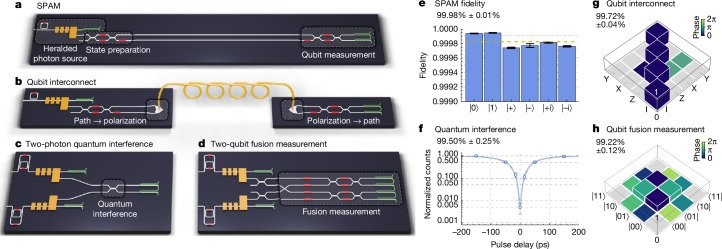
Quantum benchmarking circuits. These circuits are reconfigurable by means of thermal phase shifters indicated in red in the schematics. **a**–**d**, Schematics of: quantum state preparation and measurement (**a**); point-to-point qubit network (**b**); two-photon quantum (HOM) interference (**c**); two-qubit fusion measurement (**d**). **e**, SPAM fidelity of the reconstructed state with the target state for Pauli eigenstates. **f**, HOM interference. **g**, Measured Pauli transfer matrix^32^ of chip-to-chip qubit interconnect channel. **h**, Reconstructed two-qubit density matrix after fusion (grey bars indicate magnitude below the 0.01 threshold).

We prepare a path-encoded qubit^26,27^ using a heralded photon and two-mode interferometers, as illustrated in Fig. 3a. We measure the path-encoded qubit using a two-mode interferometer and SNSPDs. The state of the single photon in two optical modes is controlled by two thermal phase shifters, which enable the encoding of arbitrary qubit states. We observe an average SPAM fidelity of 99.98% ± 0.01% (Fig. 3e), conditional on the photon being detected (Supplementary Information). Aiming to separate the impact of the HSPS’s signal-to-noise ratio, we repeat the measurement on a different but equivalent chip, using bright coherent light and off-chip photodetectors, achieving a fidelity of 99.996% ± 0.003% (Supplementary Information), showing that higher SPAM fidelity will be possible with improved HSPS signal-to-noise ratio.

### Chip-to-chip qubit interconnect

Networking of quantum modules has seen growing interest as various technologies seek to scale beyond the boundary of a single chip, trap or reticle. Telecommunications-wavelength photonic qubits are naturally suited for transmission through optical fibre, without the need for quantum transduction^28^. Furthermore, optical-fibre-based networking can enable further new functionality, such as interleaving^29^ and active-volume compilation^30^, leading to large resource savings for fault-tolerant algorithms. To demonstrate the networking capability of our photonic qubits, we build a point-to-point qubit network (Fig. 3b) and assess the fidelity of qubits after propagating between modules. We prepare high-fidelity single-qubit states using the same qubit state preparation circuit as described above and convert to polarization encoding using a two-dimensional grating-coupler-based path-to-polarization converter^31^. We transmit the qubit over 42 m of standard telecommunications-grade optical fibre, before converting to path encoding at the receiving module and performing on-chip qubit state measurement. The transmission and receiving modules both use on-chip superconducting detectors and operate at liquid helium temperature. We determined the Pauli transfer matrix^32^ fidelity between the physical channel and the identity operation, conditional on photon arrival, to be 99.72% ± 0.04% (Fig. 3g and Supplementary Information). The system exhibits high loss associated with fibre-to-chip coupling by grating couplers (about 3 dB loss), which will be overcome in future systems using edge-coupled devices (discussed below).

### Two-photon quantum interference

To benchmark our integrated single-photon sources, we measure HOM quantum interference between heralded photons from two independent sources integrated on the same chip (Fig. 3c). The measured visibility depends on many factors, including indistinguishability, spectral purity, number purity, signal-to-noise ratio and system detection efficiency. To control these, we implement a single system that integrates the technologies described above: high-purity, tunable photon-pair sources; high extinction filter network; and high-efficiency and shielded SNSPDs.

The on-chip HOM quantum interference between heralded photons from different sources, without substantial spectral filtering, was 99.50% ± 0.25% (Fig. 3f), which—to our knowledge—is the highest measured in any platform. The experiment was performed at a pump repetition rate of 125 MHz, with a source coincidences-to-accidentals ratio of 929 ± 4, a heralded *g*^(2)^(0) = 0.00358 ± 0.00024 and a maximum Klyshko efficiency of approximately 26% (Supplementary Information).

### Two-qubit fusion

Bell fusion is a projective measurement onto two-qubit Bell states and is the prototypical example of the class of measurements that underpins the FBQC model^13^. We implement Bell fusion using type II fusion measurements^6^ on dual-rail qubits. Type II fusion uses a four-mode linear optical circuit followed by photon detection. It requires both single-qubit interference and interference between qubits, enabled by high-performance qubit preparations and high-visibility two-photon quantum interference, respectively.

We demonstrate that the fusion operation can perform a high-fidelity projection onto a Bell state, using the benchmarking circuit in Fig. 3d. Two independent path-encoded single qubits are prepared in the product state |+−⟩. Using a reconfigurable fusion-measurement network, paths are then exchanged between the qubits and the resulting state is measured through single-qubit measurements. When a photon is detected in each pair of detectors, we measure a fidelity of 99.22% ± 0.12% with the ideal Bell state. The density matrix is shown in Fig. 3h.

## Next-generation technologies

The performance of the baseline technology described above is still not sufficient for useful photonic quantum computing. In particular, silicon waveguides incur too much propagation loss for fault tolerance, photon sources require complex and power-hungry tuning and high-speed optical switching is unavoidably necessary to overcome the intrinsic non-determinism of the spontaneous single-photon sources.

We now describe some of the critical developments towards higher performance and further functionality in our next-generation-technology platforms, derived from several process flows. We focus on advanced photon sources, high-efficiency photon-number-resolving detection, low-loss waveguides, high-efficiency fibre-to-chip coupling and on-chip electro-optic phase shifters.

### Cascaded resonator source

The key performance metrics for photon sources are two-photon interference visibility and photon efficiency. However, there are other characteristics that must be addressed to enable the operation of devices at the scale of useful quantum computers. Two important considerations are the pump power required to drive the SFWM process and the thermal power dissipated at cryogenic temperatures to control and tune the source. We have implemented a cascaded resonator source that addresses these aspects simultaneously.

**Fig. 4:**
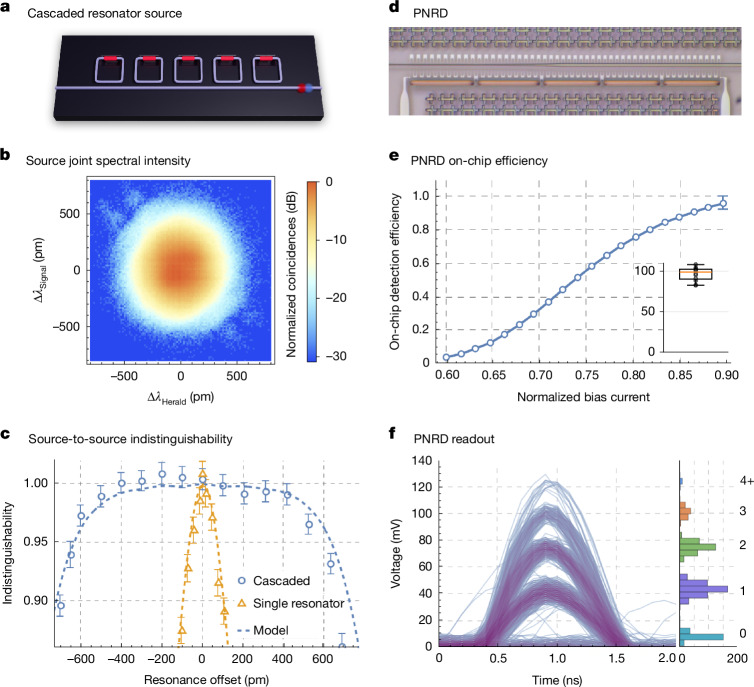
Cascaded resonator source and PNRD. **a**, Schematic of the source. **b**, Measured joint spectral intensity of a cascaded resonator source showing up to 99.35% purity, assuming flat spectral phase (Supplementary Information). **c**, Measured indistinguishability of two source copies as a function of the resonance wavelength offset (Supplementary Information). **d**, Top-down optical micrograph of a SiN-waveguide-coupled PNRD, in which single-photon detectors (SNSPDs) are crossing a waveguide and absorb light from the waveguide through evanescent coupling. Sets of SNSPDs are connected through on-chip resistors to comprise a unit cell. Identical unit cells are connected in series. **e**, On-chip detection efficiency for the PNRD shown in **d** as a function of normalized bias current, showing the average across six unique devices (Supplementary Information). **e**, Inset, distribution of single-shot detection efficiency for each of the unique devices biased at roughly 0.9*I*_SW_ at two input power levels. **f**, Left, persistent plot of the electrical photodetection signal (voltage traces) of a four-unit-cell PNRD. The traces were recorded using a cryogenic amplifier. The voltage traces show five distinct levels, corresponding to 0, 1, 2, 3 and 4 unit cells detecting photons simultaneously. Right, voltage trace histogram.

The source comprises several integrated resonators coupled to a single bus waveguide (Fig. 4a). Through joint optimization of the resonator–bus coupling, the resonance wavelengths and the pump spectral amplitude, the joint spectral intensity of the source can be engineered. Our 24-resonator device has a measured upper-bounded purity of 99.35% (Supplementary Information), assuming flat spectral phase (Fig. 4b), whilst using an order of magnitude less pump power than the interferometrically coupled source design. Even with the lower *n*_2_ of SiN, our optimized cascaded resonator source achieves 5% pair probability with approximately 100 pJ of pump pulse energy, which is within the range of scalable erbium amplifiers at GHz repetition rates.

This cascaded resonator source addresses indistinguishability in a new way. The spectrum of the photon pairs is fixed by the pump wavelength and not by the resonant wavelength of the device. Thus, global resonance shifts (for example, from fabrication variations) have minimal impact on the spectral indistinguishability of photons generated from different devices. Figure 4c shows the measured indistinguishability between two sources as a function of resonance shift (Supplementary Information). Using thermal tuners, we aligned two devices to the optimal operating point and applied a controlled global resonance shift to one cascaded resonator source, to simulate the impact of fabrication variation. In this implementation, we achieve >99% two-source indistinguishability over a ±400-pm resonance shift window, compared with less than ±40 pm for a single-ring source. The built-in tolerance of the cascaded resonator source to device-to-device global wavelength variation, together with state-of-the-art fabrication control, can enable tunerless indistinguishable photon sources.

### PNRDs

The waveguide-integrated manufacturable single-photon detectors presented earlier, although transformative, lack the photon-number-resolving capability required for FBQC. The ability to distinguish low photon numbers in detection, and to herald on that information, allows for both the removal of higher-order photon number states generated in SFWM sources and the identification of unwanted events in fusion-based entangled state generation and computation^21^.

Spatial multiplexing^33^ of many SNSPD-like detector elements, as shown in Fig. 4d, can be used to assemble a scalable detector with effective photon-number resolution. In these PNRDs, the number of detected photons is approximately proportional to the amplitude of the detector output voltage. To validate this concept, we have produced waveguide-integrated PNRDs with 4 and 5 unit cells, with the best performing designs yielding on-chip detection efficiencies of 98.9% (median) and 96.2% ± 4.3% (mean) (Supplementary Information) (Fig. 4e). These detectors have the ability to resolve 0, 1, 2, 3 and 4+ photons, as shown in the histogram of Fig. 4f (Supplementary Information).

### Low-loss SiN waveguides, directional couplers and crossings

**Fig. 5:**
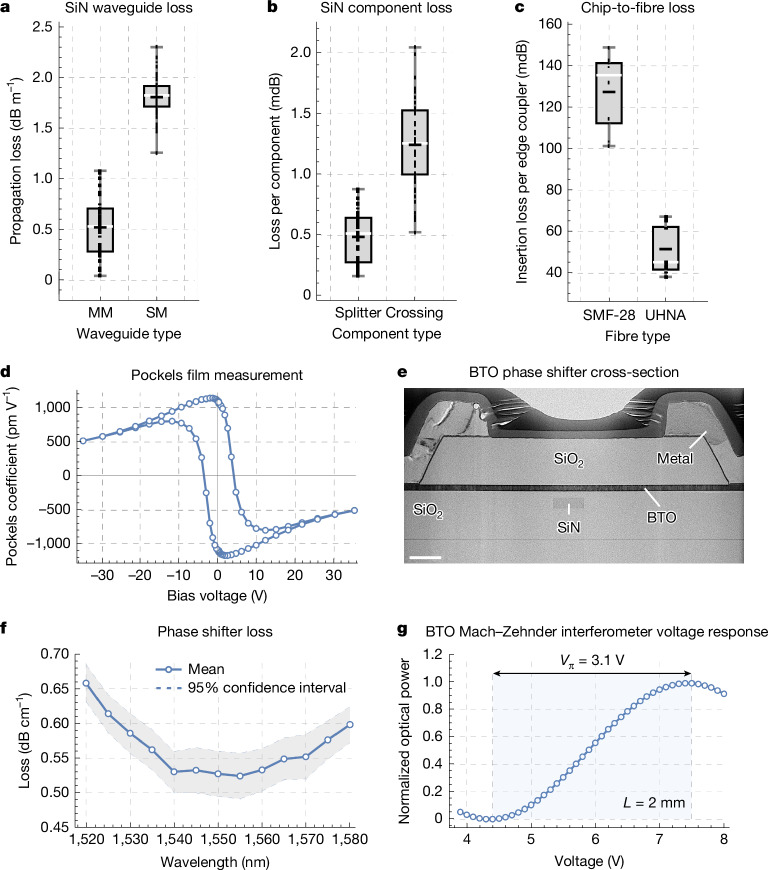
Waveguide and component loss and BTO optical switch. **a**–**c**, Loss of SiN-based components with mean (black line) and median (white line). **a**, SiN waveguide loss measurement, showing results across example wafers for both multimode (MM) and single-mode (SM) waveguides (Supplementary Information). **b**, SiN component loss for waveguide splitters and crossings (Supplementary Information). **c**, Chip-to-fibre loss. The fibre-to-chip coupling is measured in the low-loss regime using repeated transmission measurements on two exemplary devices designed for SMF-28 fibre and an exemplary device designed for UHNA fibre (Supplementary Information). **d**, Free-space electro-optic measurement of the effective Pockels coefficient of a BTO film grown by molecular-beam epitaxy, with hysteresis. **e**, Scanning electron microscope cross-section of a fully fabricated BTO-on-SiN phase shifter. Scale bar, 1,000 nm. **f**, Cutback-based propagation loss measurement of a BTO-on-SiN phase shifter (data points and guide line), with 95% confidence intervals provided (dashed lines). **g**, Measured optical transmission of a Mach–Zehnder interferometer with a *L* = 2-mm-long BTO phase shifter. A voltage was applied to one arm of the Mach–Zehnder interferometer, resulting in a *V*_π_*L* = 0.62 V.cm in a non-push–pull configuration (Supplementary Information), in which *V*_π_ is the voltage required to change the phase by π radians. Wafer maps of these results can be found in the Supplementary Information.

Silicon-on-insulator waveguides are limited in waveguide propagation loss owing to their large refractive index contrast^34^. SiN waveguides, on the other hand, have lower refractive index contrast, offering a good compromise between confinement and sensitivity to manufacturing variations^34^. We have demonstrated single-mode SiN waveguide loss of 1.8 ± 0.2 dB m^−1^ and multimode waveguide loss of 0.5 ± 0.3 dB m^−1^ (Fig. 5a), measured using a cutback technique (Supplementary Information). In this same platform, we have implemented waveguide crossings with 1.2 ± 0.4 mdB loss and waveguide splitters with 0.5 ± 0.2 mdB loss (Supplementary Information) (Fig. 5b). These component losses are about two times away from our target value, whereas the waveguide losses are on target.

SiN also provides advantages for photon generation. The ultralow loss combined with its Kerr nonlinearity supports SFWM with high signal-to-noise ratio. Further, there is an absence of nonlinear loss, allowing sources to operate with low loss at high pair rates, unlike silicon, for which two-photon absorption degrades performance^35^.

### Fibre-to-chip coupling

Low-loss coupling of light from optical fibres to our quantum photonic chips is required to make fibre networking practical. We implement new edge coupler designs that minimize mode overlap and mode conversion loss, enabling high-performance fibre-to-chip coupling. A key challenge is to convert the highly confined on-chip waveguide mode to match the much larger mode of optical fibre. To measure the insertion loss of the edge coupler, a chip is positioned between input and output optical fibres using high-precision optical alignment stages. Figure 5c shows repeated measurements from two of our best chip-to-fibre coupler designs, with coupling loss to standard telecommunications-grade optical fibre (SMF-28) of 127 ± 18 mdB and coupling loss to high-numerical aperture fibre (UHNA4) of 52 ± 12 mdB.

### Electro-optic switching

To overcome the intrinsic non-determinism of both spontaneous sources and fusion gates, photonic quantum computing will require beyond-state-of-the-art high-speed optical switches, to enable large optical networks that can be rapidly reconfigured on the basis of the results of previous heralded photon generation, entangling gates and fusion outcomes^18^. The key component required for such switching networks is a high-speed, low-loss electro-optic phase shifter. Complex *N* × *M* networks may be constructed by embedding this phase shifter into passive interferometers constructed from the beam splitter and crossing devices previously described^18^.

The performance of the phase shifter is fundamentally constrained by the choice of electro-optic material. We incorporate BTO^36^ into our photonic stack as the electro-optic phase shifter. We have developed a proprietary process for the growth of high-quality BTO films using molecular-beam epitaxy, compatible with foundry processes, on full 300-mm silicon wafers. We achieved a 3*σ* thickness uniformity of <3% across the entire 300-mm wafer, with electro-optic Pockels values of >1,000 pm V^−1^ (compared with about 30 pm V^−1^ for lithium niobate^37^), measured through free-space Pockels measurements (Fig. 5d).

The fabricated 2 × 2 BTO Mach–Zehnder switches include a 2-mm-long phase shifter section, with a propagation loss of 53 ± 3 dB m^−1^ (Fig. 5f) and a d.c. *V*_π_*L* of 0.62 V.cm (Fig. 5g and Supplementary Information). This gives a phase shifter insertion loss of about 100 mdB and a phase shifter half-wave loss-voltage product (*αV*_π_*L*) of 0.33 ± 0.02 dB.V, enabling a path to the construction of larger *N* × *M* low-loss switching networks required for photonic quantum computing. The insertion loss of this device is about two times away from our target value.

## Conclusion

We have described modifications made to an industrial semiconductor manufacturing process for integrated quantum photonics, demonstrating record performance. Through the addition of new materials, designs and process steps, we have enabled volume manufacturing of heralded photon sources and superconducting single-photon detectors, together with photon manipulation by means of interferometry, tunability and control of unwanted light. We have also described higher-performing devices, towards a resolution of the outstanding limitations of this baseline platform.

FBQC supports fault-tolerant protocols that can tolerate on the order of 10% total accumulated optical loss between photon emission and detection, with per-qubit errors in the fusion network on the order of 1% (refs. ^13,38,39^). Here we have demonstrated a feature-complete set of optical components for FBQC, each with optical losses at the several-percent or below level, as well as fully integrated circuits demonstrating high-visibility interference, distribution and measurement functionalities of photonic qubits, all with sub-percent error levels.

Improvements to the platform and processes are still required. It will be necessary to further reduce SiN materials and component losses, improve filter performance and increase detector efficiency to push overall photon loss and fidelity. Some specific examples of the remaining challenges are: implementation of low-loss *N* × *M* fast switches towards a multiplexed photon source; repeatable alignment and packaging of ultralow-loss chip-to-fibre edge connects; and improved targeting and robustness of photonic designs to minimize the need for tuning and trimming with heaters, thus further reducing the heat load at cryogenic temperatures.

Finally, we note that the platforms we have developed, and their future improvements, are highly flexible. Component arrangements are highly configurable, making the system suitable for different variations of quantum computer architectures, different quantum technology applications and, indeed, other photonic technologies. The ability to connect chips by fibre with very low loss makes the system technologically scalable across large numbers of photonic dies and allows for future networking or connections between different systems in a range of application spaces. Although the singular intent of our development is a useful fault-tolerant quantum computer, we hope that the influence of our industrially manufacturable quantum photonic platform will be broad and substantial.

## Online content

Any methods, additional references, Nature Portfolio reporting summaries, source data, extended data, supplementary information, acknowledgements, peer review information; details of author contributions and competing interests; and statements of data and code availability are available at 10.1038/s41586-025-08820-7.

## Supplementary information

Supplementary Information Supplementary Information, including Supplementary Figs. 1–22 and further references.

## Data Availability

The data that support the findings in this study are available at http://github.com/PsiQ/2404_17570.
