# Supplementary material for: A manufacturable platform for photonic quantum computing
Source: Nature. 2025 Feb 26;641(8064):876–83. doi: 10.1038/s41586-025-08820-7 (PMC12095036; doi:10.1038/s41586-025-08820-7)
Supplement: Supplementary file 1 — Supplementary Information, including Supplementary Figs. 1–22 and further references. [file 41586_2025_8820_MOESM1_ESM.pdf]

nature portfolio

https://doi.org/10.1038/s41586-025-08820-7

Supplementary information

A manufacturable platform for photonic quantum computing

In the format provided by the authors and unedited

A manufacturable platform for photonic quantum computing

Supplementary Material

PsiQuantum Team

In this appendix we provide additional details. We begin with a broader introduction to FBQC to serve as an intermediary between the introduction in the main body, and the technical published material. We then outline measurement methods and provide additional performance data on our components, circuits, and systems.

S1. FUSION-BASED QUANTUM COMPUTING

FBQC is a scheme to perform fault-tolerant quantum computing by making joint measurements—*fusions*—on qubits from distinct entangled states termed *resource states*. Regardless of the size of the computation, the size of the required initial resource states remains constant—only the total number of them grows. In previous approaches to photonic quantum computing [1–6] the probabilistic nature of entangling gates was first overcome using additional resources (e.g. for "repeat until success" offline creation of a large cluster state [7]). In FBQC, fault tolerance is hardwired into the model—the non-determinism is completely dealt with by the error correcting code at the time of computation. The replacement of huge cluster states (or ancillae for gate teleportation) by modest, fixed-sized, resource states massively reduces the complexity of building a large-scale quantum computer. A detailed description of FBQC, including analysis of resources, error correction, and computational methods, has been reported by Bartolucci *et al.* [8]. By jointly measuring one photon from each, fusions may also be used to join small entangled states together to form larger ones, and therefore as a primitive for resource state preparation. Successful fusion destroys the measured photons, but produces a single, larger, entangled state. In fact, even the small entangled states can be built from single photons using generalised fusion-type operations. Multiplexing [9–11] is used to manage nondeterminism when fusions are used in this role. Since both successful photon generation and fusions are heralded by specific detection patterns, it is possible to attempt many such operations in parallel and switch the successful cases into the machine's operational path. Fundamentally, the use of multiplexing turns an exponentially challenging scaling problem—in which multiple nondeterministic events need to all go right—to an approximately-linearly scaling challenge. A detailed description of optical switching for FBQC can be found in our other published work [12]. As the FBQC error correcting code is chosen to deal with high loss rates, it is *not* necessary that any multiplexing be performed with N so large so as to make the probability of a successful event approach unity. This, combined with the fixed size requirement of resource states, ensures that any photon only ever transverses a constant optical depth, regardless of the size of computer being built. Fusion-based architectures allow for purely ballistic operation, in which photons fly through an optical system without the need to be held in memory. Regardless of the computational size and duration, each photon exists for a limited amount of time, from source until its measurement. Entangled connectivity over time is provided by *delays* of fixed length, in which a subset of photons in one time slice are retarded (e.g. in optical fiber) and interfered (fused) with photons from a later time slice. Long low-loss fiber delays also allow photonic implementations of fault-tolerance to be extremely resource efficient. Using only a small number of long fiber links, it is in principle possible to greatly amplify entanglement generation [13]. A single generator of photonic entanglement connected back onto itself by optical fiber can usefully generate entanglement with a chain of its subsequent copies. This technique, called interleaving, can reduce the resources required to make a logical qubit by a factor of several thousand. Importantly, the exact size and form of the entangled states, and indeed some details of the fusion operations, at each of these steps are not totally constrained. There is architectural freedom to optimize these with respect to practical performance of the underlying hardware components. For this reason, targets for FT operation [8] of about 10% loss per fusion and about 1% Pauli error per fusion are indicative of performance requirements but not strictly definitive.

S2. PHOTON SOURCES

This section describes the experimental and analysis methods used for the photon pair source results reported in Fig 2b and Fig 4bc.

S2.1. Joint Spectral Intensity Measurement and Purity Estimate.

Spontaneous four-wave mixing is a broadband process. Photon pairs are generated in a superposition of monochromatic modes, according to the joint spectral amplitude $\mathcal{A}(\nu_\mathrm{h}, \nu_\mathrm{s})$ [14], where $\nu_{\mathrm{h(s)}}$ is the optical frequency of the herald(signal) photon. The joint spectral intensity $I(\nu_\mathrm{h}, \nu_\mathrm{s}) = [\mathcal{A}(\nu_\mathrm{h}, \nu_\mathrm{s})]^2$ is normalized such that $\int d\nu_\mathrm{h} d\nu_\mathrm{s}\, I(\nu_\mathrm{h}, \nu_\mathrm{s}) = 1$. The joint spectral amplitude

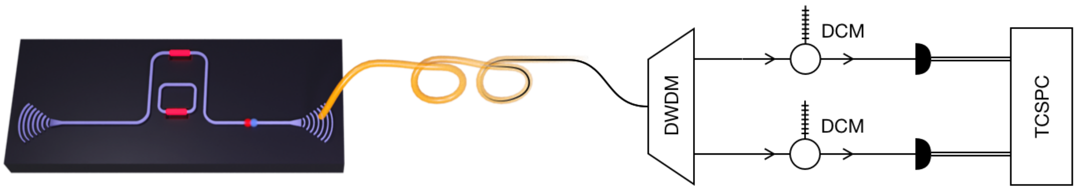

FIG. S1. Experimental setup for measuring joint spectral intensity. The measurement is performed using off-chip detectors. A fiber dense wavelength de-multiplexing filter (DWDM) blocks the pump and separates the signal and herald photons. Dispersion compensation modules (DCM) using fiber Bragg gratings introduce a wavelength-dependent delay, which is detected using time-correlated single photon counting (TCSPC).

of a photon-pair source determines the spectral purity of the heralded single photons. The amplitude can be decomposed using orthogonal normalized modes, known as Schmidt modes: $\mathcal{A}(\nu_\mathrm{h}, \nu_\mathrm{s}) = \sum_i \sqrt{\lambda_i}\, \psi_{\mathrm{h},i}(\nu_\mathrm{h})\, \psi_{\mathrm{s},i}(\nu_\mathrm{s})$, where the Schmidt coefficients λ_i are normalized: $\sum_i \lambda_i = 1$. After the detection of a herald photon, the signal photon occupies a mixture of spectral modes. With probability λ_i, the spectral mode of the signal photon is $\psi_{\mathrm{s},i}(\nu_\mathrm{s})$. Ideally, the heralded single photons are always generated in a single spectral mode. In this case, the joint spectral amplitude can be written $\mathcal{A}(\nu_\mathrm{h}, \nu_\mathrm{s}) = \psi_\mathrm{h}(\nu_\mathrm{h})\, \psi_\mathrm{s}(\nu_\mathrm{s})$, so it is a separable function of herald and signal frequencies. How close a source is to this ideal is quantified by the spectral purity, $P = \sum_i \lambda_i^2$. The spectral purity upper bounds the two-photon interference visibility that can be attained using a heralded single photon source design, as shown in the next section. Therefore, an important step in the characterization of a photon pair source is to validate the joint spectral amplitude. The measurement of joint spectral phase is possible but in this paper we only report joint spectral intensities. Assuming a flat joint spectral phase, we can extract an upper bound to the source's spectral purity.

We measure joint spectral intensity using time-of-flight spectrometers [15]. The photon pairs are coupled to fiber using grating couplers. The pump is removed and the herald and signal channels are separated using in-fiber dense wavelength de-multiplexing (DWDM) filters. For the sources discussed in the main text, the signal, pump and herald wavelengths are centered on the ITU DWDM channels 21, 33, and 45, respectively. The herald and signal channels are coupled to dispersion-compensation modules, implemented using chirped fiber Bragg gratings, adding 10ns of delay per nm of wavelength shift. The delay with respect to pump pulses is measured using superconducting nanowire detectors and time tagging electronics. The total jitter of the system is 80ps, which corresponds to a spectral resolution of 8pm. FIG 2b and FIG 4b in the main text are 2D histograms based on time-resolved coincidence measurements. The experimental setup is illustrated in Figure. S1. In the main text, we provide upper bound estimates of spectral purity extracted from JSIs, assuming flat spectral phases. The time-of-flight 2D histograms are approximations of the JSIs, affected by binning and counting noise. In our case, the resolution (8pm) and bin size (3.2pm) are much smaller than the line-widths of the measured spectra (40-100pm FWHM). Therefore, to provide purity estimates, we focus on the effect of counting noise.

Let A be the matrix constructed from the square roots of the coincidence counts versus delays. The squared singular values of this matrix are estimates of the Schmidt coefficients of the JSA. First, we verify that the data is consistent with a counting noise error model. For each bin, the measured counts are expected to be sampled from a Poisson distribution with mean proportional to the JSI at the respective frequencies. The standard deviation of the Poisson distribution with mean μ is $\sqrt{\mu}$. For large μ, the expected standard distribution of the square-root of the number of counts is $1/2$. To study the noise in the data, we use singular value decomposition to remove noise from the matrix A by replacing small singular values by 0:

$$\begin{aligned} A &= U \cdot D \cdot V^T; \\ A_n &= U \cdot \begin{pmatrix} D_n & 0 \\ 0 & 0 \end{pmatrix} \cdot V^T, \end{aligned} \tag{1}$$

where D is diagonal and D_n is the sub-matrix containing the n largest singular values. $A - A_n$ is an estimate of the noise. As illustrated in Figure S2, it is an uncorrelated random distribution with standard deviation $\approx 1/2$, which matches very well with the error model.

The effect of counting noise on the Schmidt coefficient distribution of a simulated JSA is illustrated in Figure S3. As the source purity is near-unity, the ideal matrix (without counting noise) has few large singular values. The addition of noise adds a "background" of small singular values. To estimate the effect of noise, we can add counting noise to A_n, sampling each entry from a Poisson distribution. As shown in Figure S3b, the reduction of purity due to noise converges to a fixed offset as we decrease n. Therefore, our algorithm for estimating purity is: generate counting noise on A_1 and calculate the purity, P_noise. Add $1 - P_\mathrm{noise}$ to the purity estimated from A. In the case illustrated in Figure 2b in the main text as well as Figure S2, the purity extracted from A is 0.9785. The purity from adding noise to A_1 is 0.98338 ± 0.00015. Therefore, our purity estimate is 0.99516 ± 0.00015. The error purely due to counting noise is small. However, systematic errors are expected to be higher.

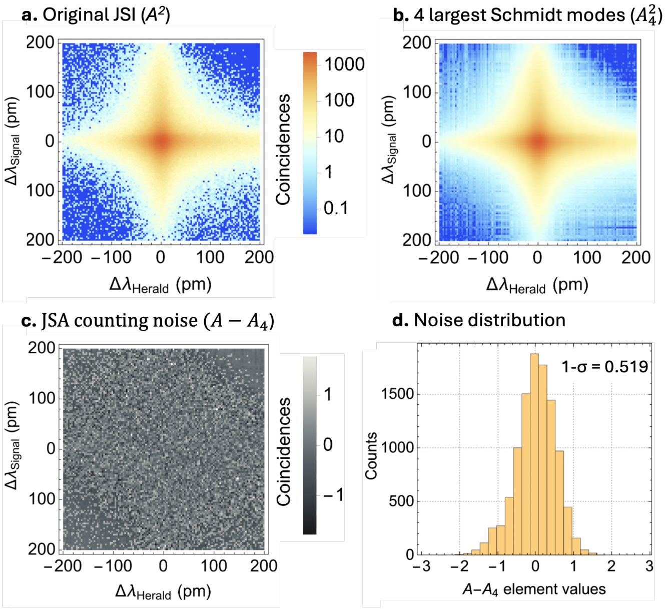

FIG. S2. **a.** Un-normalized JSI counts, using the same data as Figure 2b of the main text. **b.** The same data with noise removed by applying a threshold to the singular values. The color scale is the same as in (a.). **c.** Noise estimated by subtracting square root of (b.) from square root of (a.). **d.** Histogram of the elements of (c.). We select only the elements for which the smoothed JSI counts are higher than 1, as for regions of the JSI with sparse counts, both the noise estimation and the expectation that the standard deviation should be $1/2$ are are less applicable.

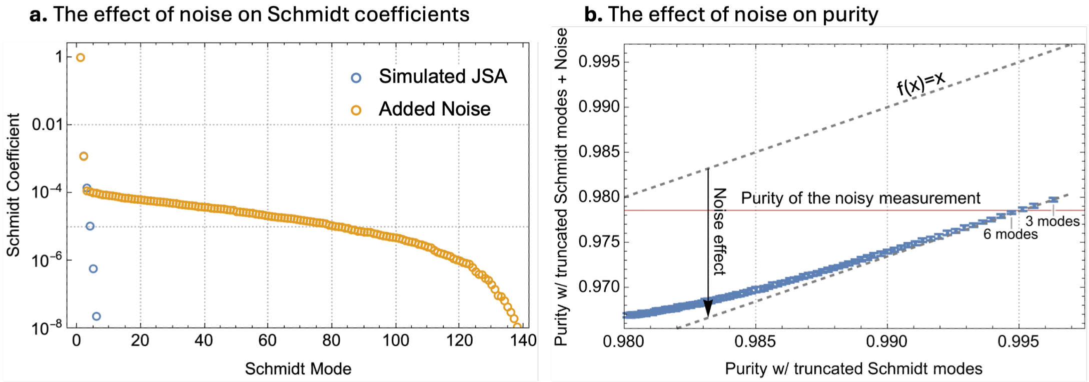

FIG. S3. **a.** Schmidt coefficients extracted from a JSA with and without noise (using a simulation of the JSA shown above). **b.** Purity obtained after adding counting noise to A_n vs. purity extracted from A_n, showing that for small n (estimating the actual JSA without noise) the effect of the noise is simply an offset on the purity.

S2.2. Source Indistinguishability Measurement

Figure 4c of the main text shows a direct measurement of the indistinguishability of two sources. Since photons are generated in single mode waveguides, distinguishability is due to their different spectral amplitudes. To measure it, the signal photons from two sources are separated on-chip and combined on a 50:50 beam splitter, as illustrated in Figure S4. The photons are collected in fibers using grating couplers and filtered using fiber DWDMs, followed by a measurement of the coincidences-to-accidentals ratio, which can be thought of as a "two-source $g^{(2)}$" or "unheralded HOM interference". To model this experiment, we first introduce the notation by considering HOM interference.

If the signal photons were heralded, the single photon states at the source outputs would be:

$$\hat{\rho}_{1,1} = \sum_i \lambda_{1,i}\, \hat{a}^\dagger_{1,i,1} |0\rangle \langle 0| \hat{a}_{1,i,1};$$
$$\hat{\rho}_{2,2} = \sum_i \lambda_{2,i}\, \hat{a}^\dagger_{2,i,2} |0\rangle \langle 0| \hat{a}_{2,i,2}, \tag{2}$$

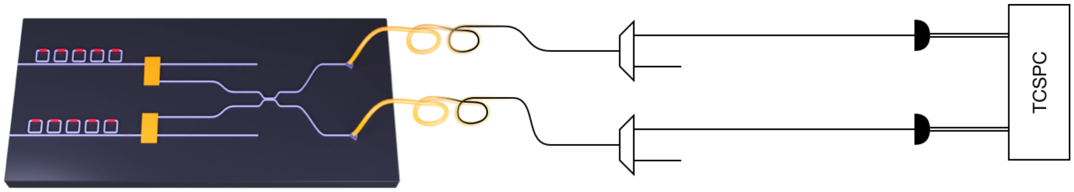

FIG. S4. Experimental setup for measuring two-source indistinguishability. The coincidences-to-accidentals ratio is measured as a function of relative time delay between the source pump pulses.

where $\hat{a}^\dagger_{1,i,1}$ creates a photon with spectral mode $\psi_{1,s,i}$ at the signal output of source 1. The first label indicates the source, and the last label indicates the spatial mode. The operators $\hat{a}^\dagger_{1,i,1}$ are broadband superpositions of monocromatic creation operators. For simplicity, we write them as discrete superpositions:

$$\hat{a}^\dagger_{1,i,1} = \sum_k \psi^*_{1,i,k} \hat{b}^\dagger_{k,1}, \tag{3}$$

where we dropped the "s" label and used the index k instead of the frequency variable for the spectral amplitudes. This representation is appropriate, as the bins can correspond to arbitrarily narrow spectral bands. The 50:50 beam splitter effectively transforms the operators:

$$\begin{aligned} \hat{U}_{\text{bs}}\, \hat{b}_{k,1}\, \hat{U}^\dagger_{\text{bs}} &= \frac{1}{\sqrt{2}} \left(\hat{b}_{k,1} + \hat{b}_{k,2} \right); \\ \hat{U}_{\text{bs}}\, \hat{b}_{k,2}\, \hat{U}^\dagger_{\text{bs}} &= \frac{1}{\sqrt{2}} \left(\hat{b}_{k,1} - \hat{b}_{k,2} \right). \end{aligned} \tag{4}$$

The probability of measuring coincidences at the output of the beam splitter is

$$p_{\text{c}} = \text{Tr}\Big[\hat{\text{U}}_{\text{bs}}\, \hat{\rho}_{1,1} \hat{\rho}_{2,2}\, \hat{\text{U}}^\dagger_{\text{bs}}\, \hat{\Pi}_{\text{c}} \Big], \tag{5}$$

where $\hat{\Pi}_c$ projects on a single photon in each output:

$$\hat{\Pi}_c = \sum_{k,l} \hat{b}^\dagger_{k,1} \hat{b}^\dagger_{l,2} \left|0\right\rangle \left\langle 0\right| \hat{b}_{k,1} \hat{b}_{l,2}. \tag{6}$$

Replacing 2, 3, and 4 in 5 and keeping only terms with one photon in each mode,

$$\begin{aligned} p_{\text{c}} &= \frac{1}{4} \text{Tr} \left[\sum_{\text{i,j,k,l,m,n}} \lambda_{1,\text{i}} \lambda_{2,\text{j}}\, \psi^*_{1,\text{i,k}}\, \psi^*_{2,\text{j,l}}\, \psi_{1,\text{i,m}}\, \psi_{2,\text{j,n}} (\hat{\text{b}}^\dagger_{\text{k},1} \hat{\text{b}}^\dagger_{\text{l},2} - \hat{\text{b}}^\dagger_{\text{k},2} \hat{\text{b}}^\dagger_{\text{l},1}) \left|0\right\rangle \left\langle 0\right| (\hat{\text{b}}_{\text{m},1} \hat{\text{b}}_{\text{m},2} - \hat{\text{b}}_{\text{n},2} \hat{\text{b}}_{\text{n},1})\, \hat{\Pi}_{\text{c}} \right]; \\ p_{\text{c}} &= \frac{1}{2} \sum_{i,j} \lambda_{1,i} \lambda_{2,j} \left[\sum_{k,l} |\psi_{1,i,k}|^2 |\psi_{2,j,l}|^2 - \sum_{k,l} \psi^*_{1,i,k} \psi_{2,j,k} \psi^*_{2,j,l} \psi_{1,i,l} \right]. \end{aligned} \tag{7}$$

Using the normalization conditions for Schmidt modes and spectral modes, and introducing the notation $\sum_k \psi^*_{1,i,k} \psi_{2,j,k} = \langle \psi_{1,i} | \psi_{2,j} \rangle$,

$$p_{\text{c}} = \frac{1}{2} \left[1 - \sum_{i,j} \lambda_{1,i} \lambda_{2,j} \left| \langle \psi_{1,i} | \psi_{2,j} \rangle \right|^2 \right]. \tag{8}$$

We drop the spatial mode label from the single photon states, letting them operate on the same spatial modes, and introduce the state overlap (using a different notation than [16]):

$$O(\hat{\rho}_1, \hat{\rho}_2) \equiv 1 - \frac{1}{2} \text{Tr} \left[(\hat{\rho}_1 - \hat{\rho}_2)^2 \right] = 1 - \frac{1}{2} \text{Tr} \left[\hat{\rho}_1^2 \right] - \frac{1}{2} \text{Tr} \left[\hat{\rho}_2^2 \right] + \text{Tr} \left[\hat{\rho}_1 \hat{\rho}_2 \right]. \tag{9}$$

Using $\mathrm{Tr}\left[\hat{\rho}_1^2\right] = \sum_{\mathrm{i}} \lambda_{1,\mathrm{i}}^2 = \mathrm{P}(\hat{\rho}_1)$ and expanding $\mathrm{Tr}[\hat{\rho}_1\hat{\rho}_2] = \sum_{\mathrm{i,j}} \lambda_{1,\mathrm{i}}\lambda_{2,\mathrm{j}} \left|\langle\psi_{1,\mathrm{i}}|\psi_{2,\mathrm{j}}\rangle\right|^2$,

$$p_{\mathrm{c}} = \frac{1}{2}\left[1 - \frac{P(\hat{\rho}_1) + P(\hat{\rho}_2)}{2} + 1 - O(\hat{\rho}_1, \hat{\rho}_2)\right]. \tag{10}$$

When measuring HOM interference, the overlap of the single photons generated by two sources is varied by sweeping their relative time delay. For a large delay, we have $\mathrm{Tr}[\hat{\rho}_1\hat{\rho}_2] \to 0$, so $p_c|\,\Delta\tau \to \inf = 1/2$. Therefore, the HOM interference visibility is

$$V_{\mathrm{HOM}} = 1 - p_{c,\mathrm{min}}/\left(p_c|\,\Delta\tau \to \mathrm{inf}\right) = \frac{P(\hat{\rho}_1) + P(\hat{\rho}_2)}{2} - \left[1 - [O(\hat{\rho}_1, \hat{\rho}_2)]_{\mathrm{max}}\right], \tag{11}$$

where $O(\hat{\rho}_1, \hat{\rho}_2)_{\mathrm{max}}$ is the maximum overlap attainable by sweeping the time delay. If the sources are indistinguishable up to a time delay, the HOM visibility is the spectral purity: $V_{\mathrm{HOM}} = P(\hat{\rho})$. $O(\hat{\rho}_1, \hat{\rho}_2)$ measures the indistinguishability of two photons. For pure sources, this is equal to the spectral overlap: $O(\hat{\rho}_1, \hat{\rho}_2) = \mathrm{Tr}\left[\hat{\rho}_1\hat{\rho}_2\right] = \left|\langle\psi_1|\psi_2\rangle\right|^2$.

Now, we can model the experiment in Figure S4. The marginal state at the signal output of a photon pair source is a mixture of thermal states [17]. If the experimental setup has efficiency η, the mean photon number of the thermal states is scaled by the factor η. We introduce the notation ε for the total photon pair generation probability times the setup efficiency. For each Schmidt mode, the state generated by the first source is

$$\hat{\sigma}_{1,i,1} = \frac{1}{1 - \varepsilon\lambda_{1,i}} \sum_n (\varepsilon\lambda_{1,i})^n \frac{1}{n!} \hat{a}_{1,i,1}^{\dagger n} \left|0\right\rangle\left\langle 0\right| \hat{a}_{1,i,1}^n. \tag{12}$$

The overall state is the tensor product $\bigotimes_i \hat{\sigma}_{1,i,1}$. As ε is a small number, we expand the coincidence probability up to the smallest order, which is ε^2. The smallest terms correspond to one photon generated in each source, and two photons generated in one source. We assume the two sources have matching single photon probabilities ε. The term corresponding to one photon in each source is $\varepsilon^2 p_c$, which we calculated above. If two photons are generated in either of the sources, the coincidence probability is $1/2$. Therefore, the term corresponding to two photon generation in either source is $(p_{1,2} + p_{2,2})/2$, where $p_{1,2}$ is the probability of two photon generation in source 1. Expanding the tensor product,

$$p_{1,2} = \varepsilon^2 \sum_{j \geq i} \lambda_{1,i}\lambda_{1,j} = \varepsilon^2 \frac{1}{2}\left[\left(\sum_i \lambda_{1,i}\right)^2 + \sum_i \lambda_{1,i}^2\right] = \varepsilon^2 \frac{1 + P(\hat{\rho}_1)}{2}. \tag{13}$$

The total coincidence probability is

$$p_{c,\mathrm{unheralded}} = \varepsilon^2 p_c + (p_{1,2} + p_{2,2})/2 = \varepsilon^2 \frac{3 - O(\hat{\rho}_1, \hat{\rho}_2)}{2}]. \tag{14}$$

We measure accidentals by counting coincidences between different pump pulses. In this case, the two outputs of the beam splitter are uncorrelated. Therefore, the coincidence probability is the product of singles probability in mode 1 and singles probability in mode 2 (either source could generate the later photon), which is simply ε^2. The result is

$$\mathrm{CAR} = 1 + \frac{1 - O(\hat{\rho}_1, \hat{\rho}_2)}{2}. \tag{15}$$

Similar to HOM interference, we sweep the relative time delay between sources. For a large delay, we have $\mathrm{Tr}[\hat{\rho}_1\hat{\rho}_2] \to 0$, so the CAR is $1 + \frac{P(\hat{\rho}_1)+P(\hat{\rho}_2)}{4}$. This is a well-known result – the marginal $g^{(2)}$ of photon pair sources is related to spectral purity [17]. On the other hand, for the highest temporal overlap, the CAR is $1 + \frac{1-[O(\hat{\rho}_1,\hat{\rho}_2)]_{\mathrm{max}}}{2}$. In practice, we optimize both the relative pump power and temporal delay to minimize the CAR.

The $g^{(2)}$ estimate of purity is susceptible to noise in the experiment. To model the noise, we introduce an additional spectral mode to the first source output, changing the state to $\bigotimes_i \hat{\sigma}_{1,i,1} \otimes (1 + \varepsilon\lambda_{\mathrm{noise}}\, \hat{a}_{\mathrm{noise}}^{\dagger} \left|0\right\rangle\left\langle 0\right| \hat{a}_{\mathrm{noise}})$. Since the experiment is unheralded, the noise will contribute to the single photon part of the state. The probability of generating a single photon in the first source becomes $\varepsilon(1 + \lambda_{\mathrm{noise}})$. The effective single photon state becomes $(1 + \lambda_{\mathrm{noise}})^{-1}\left(\hat{\rho}_1 + \lambda_{\mathrm{noise}}\hat{a}_{\mathrm{noise}}^{\dagger} \left|0\right\rangle\left\langle 0\right| \hat{a}_{\mathrm{noise}}\right)$, which changes the coincidence probability from 8 to

$$p_{c,\mathrm{noise}} = \frac{1}{2}\left[1 - (1 + \lambda_{\mathrm{noise}})^{-1}\, \mathrm{Tr}\left[\hat{\rho}_1\hat{\rho}_2\right]\right]. \tag{16}$$

The probability of generating two photons in either source becomes

$$p_{1,2,\mathrm{noise}} = p_{1,2} + \varepsilon^2\lambda_{\mathrm{noise}}. \tag{17}$$

Adding the two contributions to the coincidence probability and using 13 and 9,

$$\mathrm{CAR} = 1 + \frac{1 - O(\hat{\rho}_1, \hat{\rho}_2)}{2(1 + \lambda_{\text{noise}})}. \tag{18}$$

This shows that the experiment is Figure S4 is a simple direct measurement of source indistinguishability. The result in the main text is produced by measuring the visibility of CAR $-$ 1 while sweeping the relative time delay between sources. The result is

$$V_{\text{CAR}-1} = 1 - \frac{1 - [O(\hat{\rho}_1, \hat{\rho}_2)]_{\text{max}}}{\frac{P(\hat{\rho}_1)+P(\hat{\rho}_2)}{2}}, \tag{19}$$

such that the indistinguishability is

$$[O(\hat{\rho}_1, \hat{\rho}_2)]_{\text{max}} = 1 - \left(\frac{P(\hat{\rho}_1) + P(\hat{\rho}_2)}{2} - V_{\text{HOM}}\right) = 1 - (1 - V_{\text{CAR}-1})\frac{P(\hat{\rho}_1) + P(\hat{\rho}_2)}{2} > V_{\text{CAR}-1}. \tag{20}$$

The inequality is saturated for pure sources.

S2.3. Cascaded Array Source

The devices reported in the manuscript are manufactured in our Si photonics platform and have an free-spectral-range (FSR) of approximately 10 nm and a resonance wavelength spread of approximately 2nm. The pump linewidth, which is optimized for spectral purity, is approximately 400 pm. The resonance wavelength spread can be increased up to the FSR by adding more resonators. The resonators have identical FSR, but the resonance wavelengths are spread by design, such that a subset of the resonances overlap with the pump spectrum, leading to photon-pair generation. If all resonance wavelengths are shifted by the same amount, a different subset of the resonances will couple the pump and generate photon pairs, but their spectral characteristics remain unchanged.

The built-in tolerance of the cascaded resonator source to device-to-device global wavelength variations, together with state-of-the-art foundry process and fabrication control, can enable robust, tunerless and manufacturable indistinguishable photon sources.

S3. WAFER MAPS AND WAFER-TO-WAFER VARIATIONS

In this section we provide exemplary wafer maps to support the data shown in the manuscript. We show:

- A wafer map showing **superconducting detector performance uniformity** in the SiN process
- A wafer map showing **single-mode waveguide loss uniformity** in the SiN process
- A wafer map showing **multi-mode waveguide loss uniformity** in the SiN process
- A wafer map showing **crossing loss uniformity** in the SiN process
- A wafer map showing **splitter loss uniformity** in the SiN process
- A wafer map showing **crossing ratio uniformity** in the SiN process
- A wafer map showing **splitter ratio uniformity** in the SiN process
- A wafer map showing **BTO phase-shifter uniformity** of a BTO wafer heterogeneously integrated with SiN

Within-wafer superconducting detector uniformity: We have demonstrated, for the first time, near-unit internal detection efficiency (IDE) of SNSPDs based on NbN, with 300mm wafer-scale integration. We developed both the NbN material and the manufacturing process to achieve uniform, reproducible detector performance. Fig. S5 shows the reproducibility of superconducting single-photon detectors in our SiN-based 300mm process. Fig. S5, inset, shows the measured single photon count rate (PCR) and dark count (vortex crossing) rate (DCR) as a function of bias current. We infer the IDE by fitting a theoretical phenomenological model (a product of two error functions) to the PCR data. From these measurements we extract I_{min}, the bias current at which the inferred IDE is greater than or equal to a given target value (here, 99%), and I_{max}, the bias current at which the number of false clicks due to dark counts is larger than a given target value (here, 80cps). From these values we can compute the size of the bias operation window $I_w = I_{\text{max}} - I_{\text{min}}$ or "plateau" within which the detector can function at high efficiency with low signal-to-noise. Fig. S5 shows PCR curves for 80 unique devices across 20 dies of a 300mm wafer, and shows I_w for each die. A large bias operation window is achieved for all devices and all dies. We show the performance of our earlier generation Si-Platform results we show in manuscript later in Fig. S10.

Within-wafer SiN passive component uniformity: Fig. S6 shows the observed within-wafer uniformity of various figures of merit of passive (silicon nitride) components. We show single-mode waveguide propagation loss, multi-mode propagation loss, splitter loss and ratio uniformity, and crossing loss and ratio uniformity.

Wafer-scale uniformity of heterogeneously integrated BTO: Fig. S7 shows uniformity of BTO phaseshifters. This data is taken from 300mm epitaxial BTO grown via molecular beam epitaxy. This BTO is heterogeneously integrated via oxide bond together with 300mm silicon nitride from GlobalFoundries. This data represents 95% functional yield after hybrid integration, with average $V_\pi L$ of 0.77 $\pm$ 0.04 V.cm.

Wafer-to-wafer uniformity The above figures describe within-wafer uniformity. Part of the reason that we choose to work with mature semiconductor tools and foundry processes is to access good wafer-to-wafer uniformity. In our Gen1 (Si) process, we achieve wafer-to-wafer uniformity in the NbN nanowire width (1-sigma) of 1.6nm. In the same process, we achieve wafer-to-wafer uniformity (1-sigma) of nanowire thickness better than 0.1nm. In our Gen2 (SiN) process, we achieve wafer-to-wafer thickness uniformity (1-sigma) of the SiN film better than 0.7nm. We have measured wafer-to-wafer refractive index uniformity (1-sigma) of 0.01%. This meets our technology requirements for SiN waveguide performance.

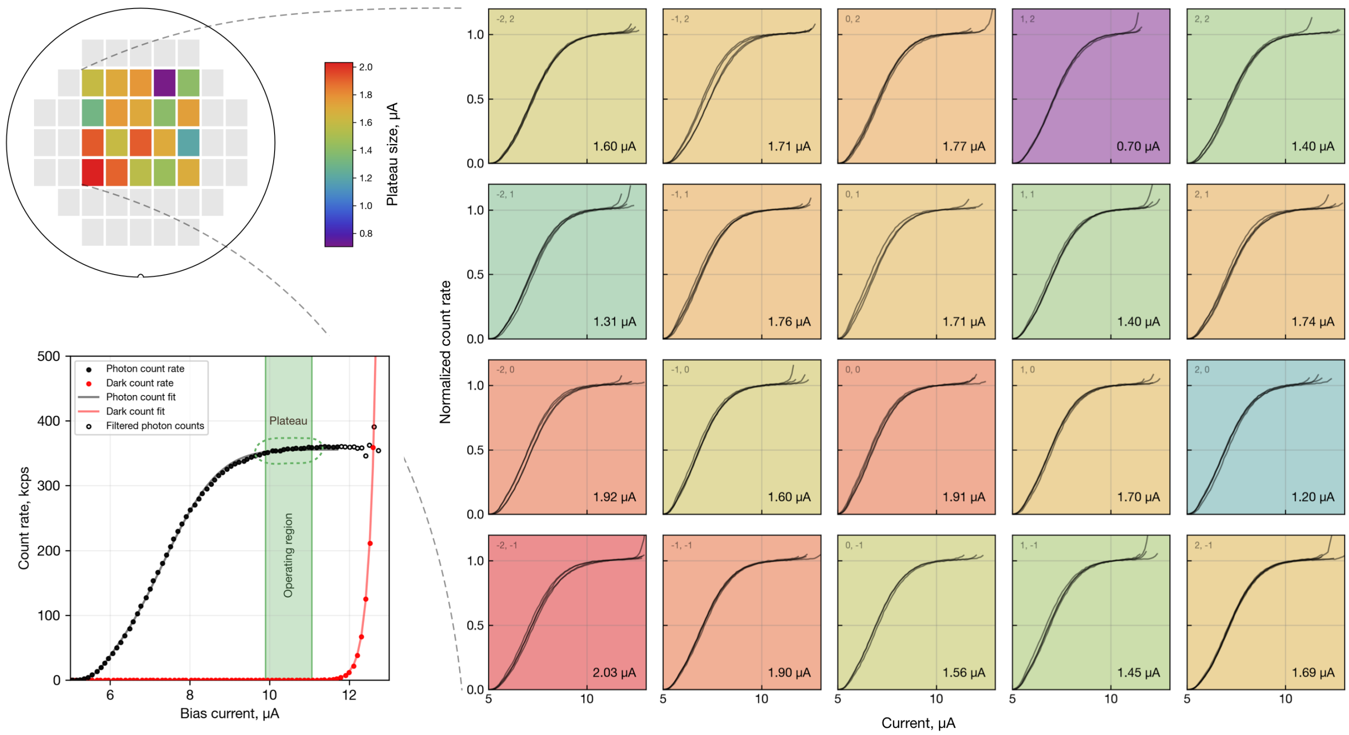

FIG. S5. **Within-wafer superconducting detector performance.** Within-wafer superconducting detector performance. Above 99% IDE (Internal detection efficiency) has been robustly achieved in a large operation bias current regime on a wafer scale. The plateau size, illustrated in the main plot of count rate vs. bias current, is defined as the range in bias current at which the IDE is larger than 99% and the dark count rate is low (shown in a green overlay). Individual measurements of 80 unique devices over 20 dies show consistent plateau size between 0.7 to 2uA per die. Each subplot shows the data for four devices per die (die coordinates are noted in the upper left corner), with the mean plateau size noted in the bottom right. The high performance and uniformity of this wafer (shown as a colormap overlayed on the wafer map) is such that these detectors can be operated in the high-efficiency regime with a single choice of bias current, i.e., without tailoring bias current to each device.

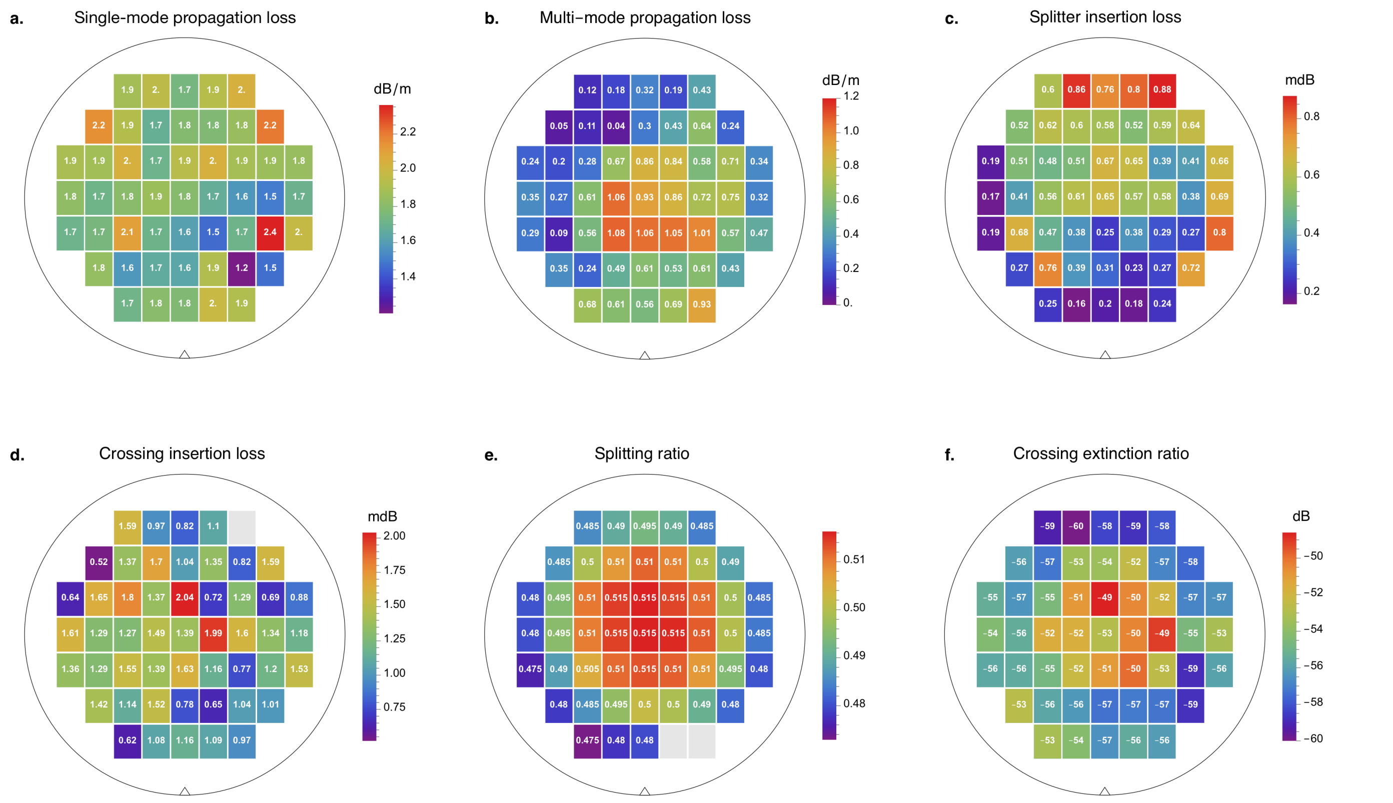

FIG. S6. **Within-wafer SiN passive component uniformity**. Representative wafer maps showing **a**, within-wafer uniformity of silicon nitride single-mode waveguide propagation loss **b**, within-wafer uniformity of silicon nitride multi-mode (routing) waveguide propagation loss **c**, within-wafer uniformity of per-component splitter (directional coupler) loss **d**, within-wafer uniformity of per-component crossing loss **e**, within-wafer uniformity of splitter (directional coupler) splitting ratios **f**, within-wafer uniformity of per-component crossing extinction ratio.

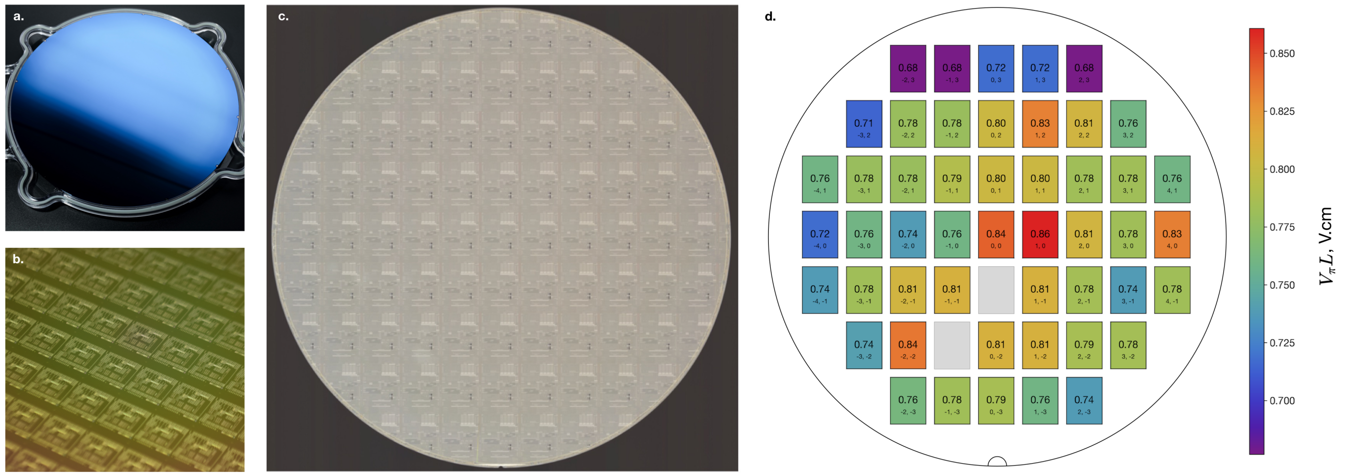

FIG. S7. **Wafer-scale uniformity of heterogeneously integrated BTO. a.** A 300mm wafer of epitaxial BTO. **b.** Optical switches fabricated using PsiQuantum's 300mm SiN process and 300mm epitaxial BTO. **c.** Wafer-level optical image of a 300mm wafer of BTO devices, after hybrid integration. **d.** Wafer map showing measured uniformity of $V_\pi L$ for the finished wafer. We observe 95% functional yield, average $V_\pi L$ of 0.77 ± 0.04 V.cm and maximum (worst-case) $V_\pi L$ of 0.84 V.cm.

S4. PHOTON DETECTION

We provide a schematic of the PNRD in Fig. S8.

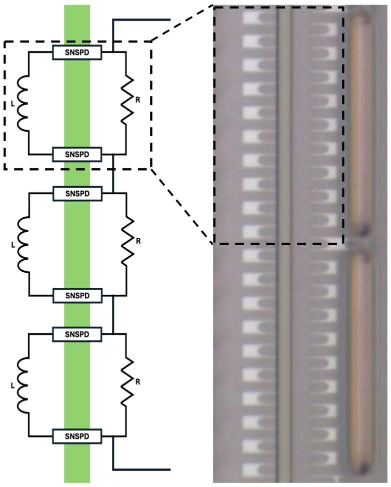

FIG. S8. **Schematic representation of a waveguide-integrated PNRD.** The PNRD comprises multiple unit cells - in this sketch, 3 unit cells are shown. The PNRD in this work comprises 5 unit cells. Each unit cell is composed of one SNSPD (photosensitive nanowires crossing a waveguide) connected electrically in parallel to a resistor R. We call out the distinct photosensitive areas of a single SNSPD with unique SNSPD labels in the diagram, noting that these are constitute parts of a single SNDPD. The inductance L required to avoid latching is provided by the total kinetic inductance of the SNSPD. Multiple identical unit cells are connected electrically in series to form a PNRD.

S4.1. Detector on-chip efficiency measurement setup and procedure

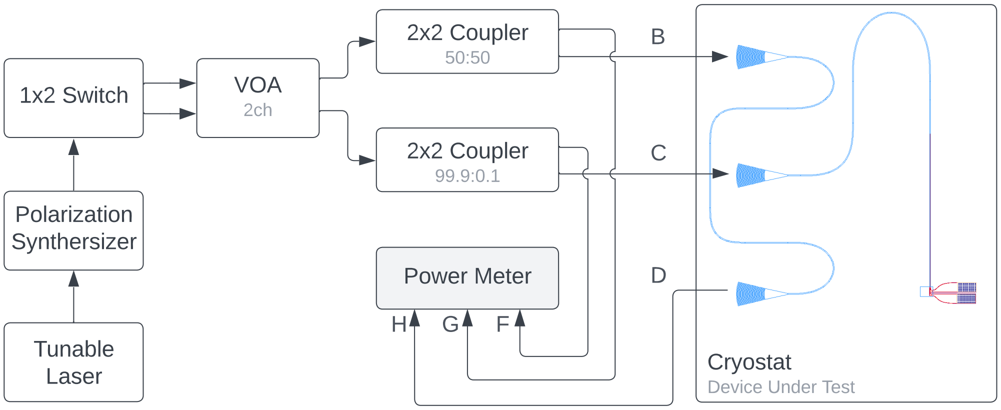

FIG. S9. Optical setup used for on-chip efficiency measurements. The output of a tunable laser is connected to a polarization synthesizer before going to an optical switch. The switch can be configured to direct the light into the loopback (top) path or the detector (bottom) path. The loopback path consists of a variable optical attenuator (VOA) followed by a 50:50 optical coupler that splits half of the light to the device (B) and half to one of the channels of an optical power meter. The detector path consists of a VOA followed by a 99.9:0.1 optical coupler that splits a fraction of the light to the device (C) and the majority of the light to one of the channels of an optical power meter. The uneven splitting ratio is necessary to deliver single photon regime light to the detector, while maintaining a monitor power well above the noise floor of the power meter.

The on-chip efficiency of waveguide-integrated SNSPDs and PNRDs is characterized by coupling light into the optical input of the chip (in this case grating couplers) through an optical fiber array unit (FAU). The FAU is placed inside the cryostat

positioned and mounted on top of cryogenic piezo stages to actively align to an array of grating couplers. The FAU is aligned to an array of 4 grating couplers, labelled as B, C, D in Figure S9 which schematically shows the optical setup. Two optical paths are used for characterizing the on-chip detection efficiency of single-photon detectors: The detector-coupled path (C) and the reference path (B, D) used to calibrate the optical coupling losses into the chip. During the measurements the electrical output of the detector is connected to amplifiers which feed the electrical signal from the detector (photodetection pulses) into a pulse counter.

The measurement procedure while the detectors are at base temperature ($\sim 2.1K$) is as follows:

1. Leave laser off and close all physical shutters in the optical path. Measure the cryogenic I-V curve of the detector and extract the switching current (Isw) of the detector, which is the maximum current at which the detector is superconducting.
2. Bias the detector at 90% of I_{sw} and sweep the voltage threshold of the electrical pulse counter to determine the minimum voltage threshold of the pulse counter above the electrical noise floor.
3. Set 1x2 optical switch to detector path (going through VOA 1), turn on the laser, keep detector biased at 90% of I_{sw} and set threshold to the optimal value found in (2.). Set attenuation of the variable optical attenuation to mid-scale and sweep the polarization states of the polarization synthesizer to find the optimal polarization state (SOP) that maximizes the observed count rate from the detector.
4. While the detector is biased at 90% of I_{sw}, the counter is set to the optimal value found in (2.) and the polarization synthesizer set to the best SOP found in (3.), sweep the optical attenuation and measure count rate to find the maximum attenuation with the detector still operating in single-photon regime. When the detector is in single-photon regime, the observed detector photon count rate is linear with optical power.
5. Using all settings from (4.), and the best optical attenuation setting found in (4.) to ensure detector operation in single-photon regime, sweep the detector bias current from 60% to 100% of I_{sw} and measure count rate with light on (CR(Ib)) and with light off (DCR(Ib)). Record the monitor detector waveguide input power.
6. Set 1x2 optical switch to the loopback path (going through VOA 2). Set the switch to the loopback path, set the optical attenuation to 0 attenuation (maximum power) and sweep the polarization states of the polarization synthesizer to find the optimal polarization state (SOP) that maximizes the power transmitted through the loopback. Extract the loopback output and the monitor input power at best SOP to calculate loopback loss.
7. Estimate power in the waveguide by correcting the monitor powers by the calibrated correction factors of the optical couplers and subtracting half of the loopback loss (under the assumption that the loss (fiber + grating coupler) of each channel of the fiber array and the loss of the GCs are the same by design)
8. Calculate efficiency (Ib) by getting the PCR(Ib) = CR(Ib)-DCR(Ib) and normalizing by the estimated photon flux in the waveguide, that is the estimated power from (7) divided by the energy of the photon (at 1550nm)

Note1: that the best SOP for detector path and loopback path need to be optimized independently because the physical fibers are different, and they can introduce different polarization rotation. Note2: we measure the efficiency at different attenuations to verify we are in single photon regime and count scale linearly with power and there is not undercounts/overcount effect. Details on estimation of power in the waveguide below.

C1 and C2 are the calibration values of the coupling ratio of the two optical couplers, used in the test rack to create a monitor channel of the light sent to the fiber array.

S4.2. Calculation of average on-chip detection efficiency from dataset

Here we give a summary of the populations of detectors presented in the main body, and in the Figures S11 and S10.

Si-waveguide-integrated superconducting nanowire single-photon detector (SNSPD):

- single-mode Si waveguide integrated hairpin-shaped superconducting nanowire single photon detectors (SNSPDs)
- absorption in a less photosensitive portion of the hairpin (U-bend) limited efficiency in this design.
- on-chip efficiency for 4 dies measured from best process split. Measured at $\sim$ 2.1 Kelvin.
- 4 dies, 12 devices per die, 48 devices total. 11 devices had bad measurements (e.g. electrical noise or probing issues) or broken devices (excluded from efficiency stats) and were excluded.

SiN-waveguide-integrated Photon Number Resolving Detectors (PNRD):

- PNRDs comprising 100 photosensitive nanowires crossing a SiN waveguide. Subsets of 20 nanowires are connected into unit cells, resulting in 5 unit cells total
- on-chip efficiency for 2 dies measured for best process split. Measured at 2.1 Kelvin.
- 2 dies, 3 devices per die, 2 power setpoints for each device (53-54 dB & 62-63 dB), 12 measurements total, all included in efficiency stats.

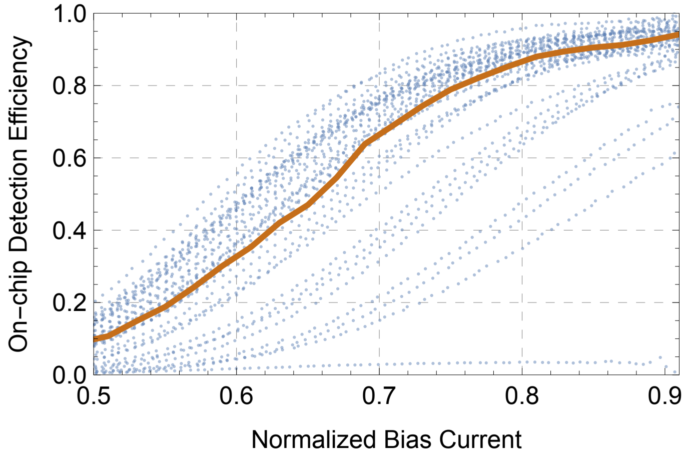

FIG. S10. Efficiency versus bias current of the 38 Si-waveguide-integrated SNSPDs. A median on-chip efficiency of 93.4% and average value of 88.9% $\pm$ 3.5% were obtained by extracting the efficiency number at the 90% of I_{sw} bias current (or the measured bias current closest to 90% of I_{sw})

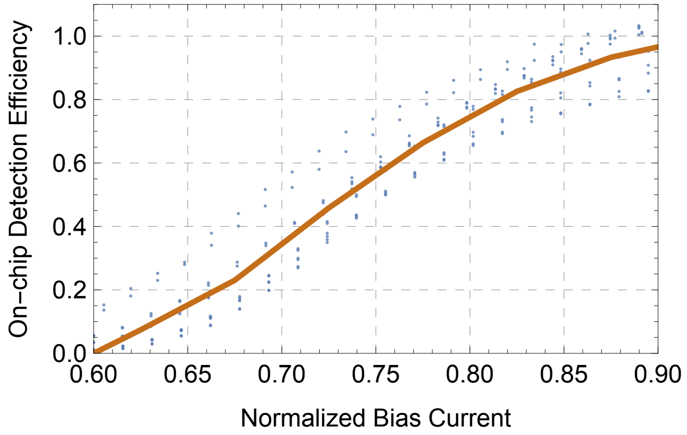

FIG. S11. Efficiency versus bias current of the 6 SiN-waveguide-integrated PNRDs at 2 different attenuations levels (dotted lines) and the averaged efficiency across all the devices (solid line). Showing 98.9% (median) and 96.2 $\pm$ 4.3% (mean) at the device operating point. The average curve has been obtained by averaging the efficiency values of different devices and attenuations at the same normalized bias current. Note that since a fixed voltage trigger level was used to record/count the PNRD pulses, at bias current values below $\sim 0.8\, I_{\mathrm{sw}}$ some PNRDs suffer from lower signal-to-noise ratio, which we believe contributes to missed pulses and suppressed efficiency below $\sim 0.8\, I_{\mathrm{sw}}$. Additionally, since the devices have some variability in the I_{sw}, the normalized bias points do not overlap perfectly, and to compute the average efficiency the relative bias currents are grouped to the closest value amongst the different devices. An alternative would have been to interpolate all the curves and to extrapolate efficiency values. The difference between the two methods is expected to be well below the measurement error bar and the preference was to use only measured values and not extrapolated ones.

S4.3. Measurement uncertainty estimates for on-chip detection efficiency

The main contributions to the uncertainty of our measured on-chip detection efficiency are: calibration, alignment uncertainty between the fiber array and the device, grating coupler variability, and detector variability.

The setup calibration uncertainty is common for all measurements, while the grating coupler and detector device variability depend on the device type and material stack under test. The error estimates quoted in this section are referring to $\pm 1\sigma$ estimates.

Calibration uncertainty: The setup calibration uncertainty is estimated to be about 2.5%. This is the results of the quadradic sum of the contributions are outlined below.

Calibration of optical losses and splitting ratios in the measurement chain: This contribution is estimated to be 1.4%, dominated by polarization dependent loss (PDL) in our optical splitters (of about 1% each).

Stability of laser and optical chain: The stability of laser and optical chain (excluding the device under test) was measured and estimate to contribute less than 0.01% uncertainty to detector efficiency. We also verified the stability of the total optical path through the on-chip loopback to contribute less than 0.1% uncertainty.

Power meter calibration: After the measurements in this paper were obtained, we had two of our optical power meter channels calibrated at NIST, revealing systematic offsets of 1.4% and 2.2%. This suggests an overall uncertainty in the efficiency measurements of around 2%. In future measurements using the calibrated channels, this factor will be reduced to the uncertainty of the calibration, about 0.2%.

Alignment variability: We use cryogenic nano-positioners to align a fiber array to our grating couplers. Our alignment procedure involves fixing sample and fiber array angles once per die and realigning the translational stages of the fiber array for each device. By repeating both procedures multiple times for the same set of devices, we have found that the dominant source of alignment-related measurement uncertainty comes from our sample angle optimization procedure. This contributes about 3% uncertainty to detection efficiency.

Grating coupler loss variability: We use 3 different grating couplers for the OCDE measurements: one grating coupler to deliver light to the detector and the other two to estimate the grating coupler loss. Variation from grating to grating adds uncertainty to our measurement. In addition to loss, variation in polarization dependence also contributes to uncertainty. The grating coupler variability can be reduced by averaging measurement results from identical copies of devices. The grating coupler variability has been estimated by measuring loopback loss on many devices across a die. From this dataset, we calculate the difference in loopback loss between each pair of loopbacks and look at the distribution of those differences as a function of the distance between them. We extrapolate this function to the distance between the grating couplers used for the OCDE measurements to estimate the variability of adjacent grating couplers.

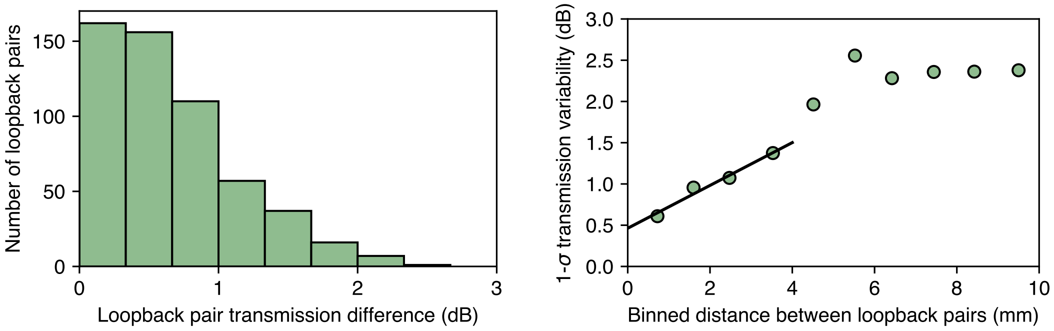

FIG. S12. SiN grating coupler variability data. Left: histogram of optical transmission differences between loopback pairs separated by less than 1.5 mm on a die. Right: The 1-sigma (encompassing 68% of the data) width of the distribution as a function of distance between the loopback devices. Below 4 mm separation, there is a linear trend, allowing us to infer 0.5 dB variability between adjacent gratings (127 μm apart). This translates to roughly 11.5% variability, contributing 5.75% uncertainty to our efficiency estimation.

Si grating couplers: For Si-waveguide-integrated SNSPDs, the grating coupler variability was estimated to be 2%.

SiN grating couplers: For SiN-waveguide-integrated PNRDs, the grating coupler variability was estimated to be 5.75% (see figure S12). We also investigated differences in optimal polarization between neighboring grating couplers and found this to add about 0.2% uncertainty.

Other sources of uncertainty: We also evaluated other sources of uncertainty and found their contributions to be much smaller than the other factors discussed above. These included laser wavelength uncertainty, scattered light, afterpulsing, electrical triggering uncertainty, photon shot noise, and dark counts. Due to triggering uncertainty and device variability below $\sim 0.8\ I_{\rm sw}$, we limit the error estimates to the operation regime close to $\sim 0.9\ I_{\rm sw}$.

Calculation of total uncertainty: Single shot efficiency errorbar is obtained by summing quadratically the contribution of the main sources of variability. This errorbar is relative to the OCDE value, therefore the absolute value of the errorbar needs to be multiplied by the efficiency at the specified bias point. When averaging efficiency from different devices, the grating coupler variability can be reduced by the square root of the number of averaged devices.

Si-WG-integrated SNSPDs: The quadratic sum of the optical setup calibration uncertainty, alignment variability and grating coupler variability is $\sqrt{2.5^2+3^2+2^2}=4.4\%$. The averaging of the 37 SNSPDs reduces the grating coupling variability from

2% to 0.3%. This reduces the total quadrature error to 3.9%.

We compared the estimated single shot measurement uncertainty of 4.4% to the variability of the OCDE dataset at for the 37 devices under test at the operation point (90% of I_{sw}). The variability of the OCDE dataset is $\sim$ 3.8% (after excluding 4 outlier low-efficiency devices), which is comparable to the estimated single shot measurement uncertainty.

SiN-WG-integrated PNRDs: The quadratic sum of the optical setup calibration uncertainty, alignment variability and grating coupler variability is $\sqrt{2.5^2 + 3^2 + 5.758^2} = 7\%$. The averaging of the six PNRDs can reduce the grating coupling variability by a factor of $\sqrt{6}$, resulting in a total relative errorbar of 4.5%.

We compared the estimated single shot measurement uncertainty of 7% to the variability of the OCDE dataset at for the 6 devices under test at the operation point (90% of I_{sw}). The variability of the OCDE dataset is $\sim 6.2\%$ (after excluding 1 outlier low-efficiency device), which is comparable to the estimated single shot measurement uncertainty.

S4.4. Number resolution

To demonstrate and test the number resolving capability of the detector, we model the correspondence between input state and "click outcome". In practice, this correspondence describes the relationship between individual peaks in the PNRD readout and distinct photon number events, and can be tested by illuminating the PNRD with coherent laser light. Under uniform illumination with coherent light, the probability of observing a given click outcome is modelled by a single parameter distribution. We derive this distribution parameter, $\tau = 1 - (1 - p_{\mathrm{dc}} e^{-\frac{\eta_{\mathrm{eff}}}{F}|\alpha|^2})$, where $|\alpha|^2$ is $\langle n \rangle$ of the illuminating coherent state, $|\alpha\rangle$, p_{dc} and η_{eff} are unit cell dark count probability and internal efficiency, and F is the PNRD fan out size or number of unit cells.

We test a 10 unit cell device by first illuminating it with pulsed laser light at a repetition rate of 20 MHz. From the voltage signal recorded on a fast oscilloscope, the maximal value of the trace within a 1 ns window around the trigger is recorded. The peak values are histogrammed, from which multiple discrete response bands (click outcomes) can be identified, as shown in Figure 4f in the main text.

This is repeated for multiple pulsed laser attenuations, and each distribution is fit to the model distribution. The observed scaling of τ with respect to input attenuation is reflective of the efficiency of the device, and the linear scaling with attenuation is consistent with our model, confirming that "clicks number" correspond to photon number as expected. The measurement is repeated for different biases, i.e. different internal efficiencies, which induces a change of the distribution parameter, since detection inefficiency and input attenuation are not separable. We also observe the corresponding variation in dark counts extracted from the model as a function of varying bias.

An indication of uncertainties is derived from sensitivity to the bin edge position. Thresholds between the levels are initially estimated via kernel density estimation, with a scale factor commensurate with the level widths; from this, the relative occurrence of click numbers can be estimated. Changes in the obtained number distributions when resampling the position of the "click number" thresholds over a lengthscale comparable with the histogram bin size; where, the long(short) error bars in the histograms, S13 left, indicate extremal(quartile) counts observed when different bin edges are used. Each of the resampled histograms are independently fit to the model distribution, S13 right, and the spread of the obtained distribution parameter are correspondingly used to estimate error bars.

The model is generalizable to the case of a waveguide coupled device, and–in combination with additional measurements–can allow an estimate of the conditional probabilities.

S5. SIN WAVEGUIDE AND PASSIVE COMPONENTS LOSS ESTIMATION

We measure device insertion loss using the standard cutback method, where multiple copies of the device are connected in series in increasingly large sets. We refer to these sets as cutback elements. Using an array of optical fibers aligned to grating couplers, laser light is injected into the input of each cutback element and measured at the output using an optical powermeter. The optical power is expected to decrease when the light propagates through a larger number of devices. Therefore, by measuring the additional optical loss introduced by larger cutback elements and assuming this loss is proportional to the number of devices in the light path, we can estimate the insertion loss per device.

For waveguide loss, we use three waveguide spirals as the cutback elements. The input-output routing and the bends, which are necessary for tightly packing long paths in a small area, are identical for the three spirals, as illustrated in Figure S14. The difference between them is the length of the straight waveguide sections. In total, our additional straight waveguide lengths are $\Delta \mathrm{L}_1 = 0\,\mathrm{cm}$, $\Delta \mathrm{L}_2 = 20\,\mathrm{cm}$, and $\Delta \mathrm{L}_3 = 40\,\mathrm{cm}$. The data we collect is the input-output optical loss through the 3 spirals, measured in dB. This includes losses through the test system, the grating couplers, the routing and bends of the waveguide spirals, and the straight waveguide lengths.

The model used is based on the following assumptions: losses are additive, the loss of the test system, the gratings, routing, and waveguide bends have no systematic variation between the 3 devices, and the straight waveguide sections introduce a

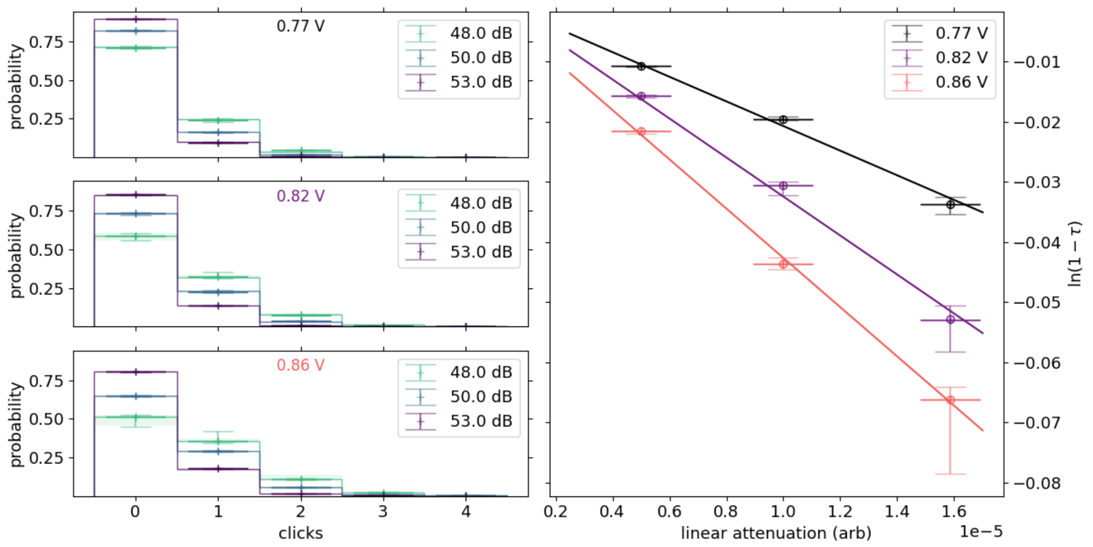

FIG. S13. PNRD performance: (left) Measured outcome probability as a function of input illumination, where we vary the attenuation of the probe, repeated for increasing bias currents (top-bottom). The points indicate the estimated probability of click events while the bars display the fitted probability distribution. Error bars on the points are derived by perturbing the bin discrimination thresholds, and re-allocating marginal events: peaks with a higher uncertainty which underlying click outcome these correspond to. The corresponding variation in fitted distribution is shown by the shaded area in the histogram steps. (right) variation of the fitted distribution parameter, τ, for each of the curves on the left, as a function of illumination amplitude, fit to a linear model. For differing bias currents, the resulting change in efficiency is manifest in the varied $\frac{\mathrm{d}\tau}{\mathrm{d}\alpha}$.

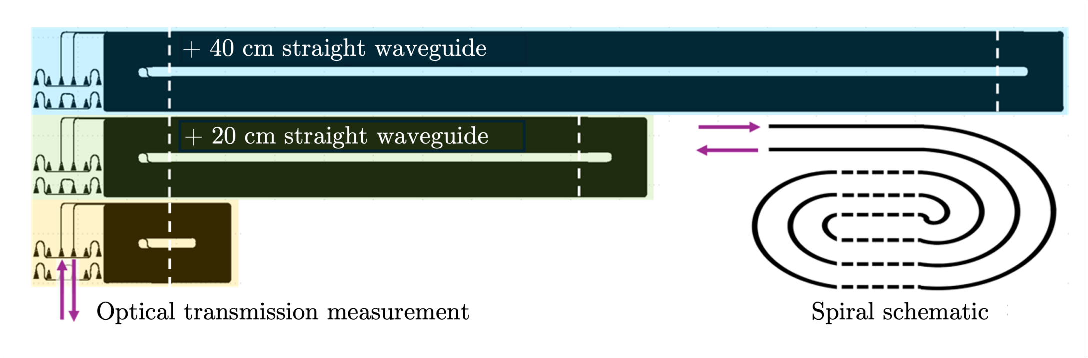

FIG. S14. Cutback design for waveguide loss estimation.

loss proportional to their total length. The waveguide propagation loss is the proportionality constant between the straight waveguide's loss and its length. We estimate this parameter, which we can use to predict photon losses in circuits and systems. To ensure that the assumptions enumerated above are valid, we designed the waveguide bends in the spiral such that they produce negligible coupling between the fundamental waveguide mode and other supported modes. Another possible systematic error can result from light scattering into propagating modes other than the fundamental forward-propagating mode due to waveguide wall roughness. We ensure that this effect is negligible by verifying that the measured optical loss differences are indeed proportional to the straight waveguide length differences and by verifying that the optical transmission through the spirals has a smooth spectrum without significant oscillations or sharp features. These add noise (not systematic errors) to the loss estimates at a given wavelength.

The model for our parameter estimation problem is:

$$\Delta \mathrm{IL_{i,j}} = \mathrm{IL_{i,j}} - \mathrm{IL_{1,j}} = \alpha \Delta \mathrm{L_i} + \delta \mathrm{IL_{i,j}}. \tag{21}$$

Here, $\mathrm{IL_{i,j}}$ is the measured optical loss for spiral i in cutback j. $\mathrm{IL_{1,j}}$ is used as a reference for each cutback, accounting for the loss of the measurement system, grating couplers, routing and bends. $\delta\mathrm{IL_{i,j}}$ accounts for random insertion loss variations, and has an expected value of 0. In our measurements, these variations are dominated by the grating couplers. Finally, α is propagation loss, the parameter we aim to estimate.

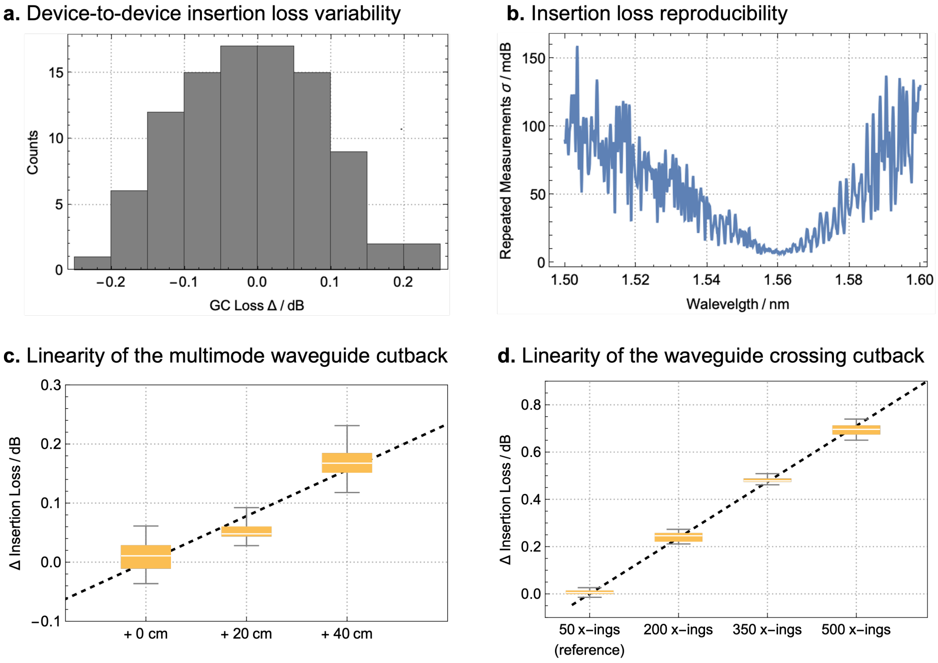

FIG. S15. **a.** Distribution of the insertions loss difference between grating coupler loopbacks within a cutback. The loop-backs are placed below the cutback element grating couplers, as shown in FIG S14. The insertion loss uncertainty has a standard deviation on the order of $0.1\,\mathrm{dB}$. **b.** Variability of repeated measurements on the same device, estimated from measuring all cutback elements 10 times accross a wafer. **c.** Box chart showing all insertion loss measurements for the multimode waveguide result reported in the main text. **d.** Box chart showing all insertion loss measurements for the waveguide crossing result reported in the main text.

Assuming $\delta\mathrm{IL}_{\mathrm{i,j}}$ is normally distributed with standard deviation σ, the maximum likelihood estimate for α is equivalent to the slope of the min-squared-error linear fit of insertion loss versus propagation length. For our lengths, the maximum likelihood estimate and the expected standard deviation of the likelihood function are:

$$\begin{aligned} \alpha_{\mathrm{ML}} &= \left\langle \frac{\mathrm{IL}_{3,\mathrm{j}} - \mathrm{IL}_{0,\mathrm{j}}}{40\mathrm{cm}} \right\rangle; \\ \sigma_\alpha &= \frac{1}{\sqrt{2m}} \frac{\sigma}{40\mathrm{cm}}, \end{aligned} \tag{22}$$

where the average is over all cutbacks and m is the number of cutbacks measured. As illustrated in FIG S15a, σ is on the order of $0.1\,\mathrm{dB}$, so we can estimate propagation losses above $0.2\,\mathrm{dB/m}$ from a single cutback measurement. For crossings and directional couplers, we use the same cutback method, with 50, 200, 350 and 500 devices in the cutback elements. The directional couplers have both output ports of each copy connected to the two input ports of the subsequent device. The cutback elements have two outputs. We measure the optical power in both outputs and sum the results to obtain the optical loss.

For each loss figure reported in the main text, we report the estimated α at $1560\,\mathrm{nm}$ from cutback measurements across a wafer. The reported error is the device-to-device standard deviation across the wafer. The figures reported are not all from the same wafer or from the same process. However all the processes used are stable and reproducible, using $350\,\mathrm{nm}$ thick SiN waveguides, and we expect to integrate the reported performance and further improvements into our the next generation quantum photonics platform.

S6. FIBRE-TO-CHIP OPTICAL COUPLING

A carefully designed edge coupling procedure is necessary to verify low-loss performance of exemplary devices. The measurements use an active optical alignment setup (Fig.S16) with a CW laser source and photodetectors to measure transmission through the chip with optical fiber probes. An accurate measurement of the initial system loss (losses of the auxiliary fiber optics and equipment that should not be included as part of the edge coupler loss) must not involve disconnecting reconnecting fiber connectors, as fiber connectors can have a large loss repeatability error. Instead, the system loss is first measured with an

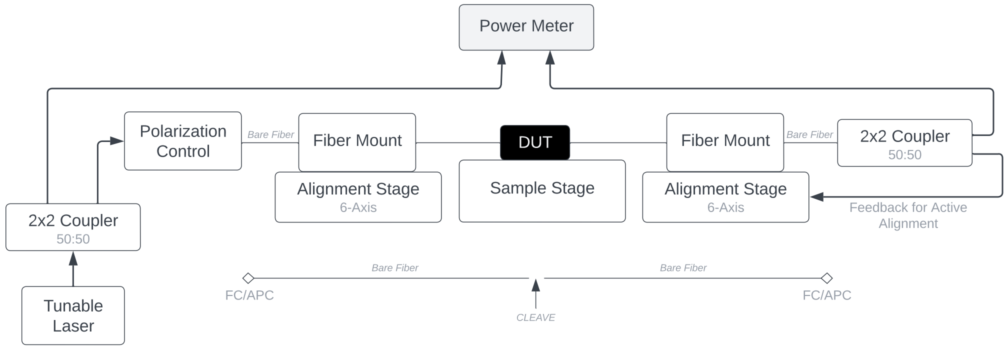

FIG. S16. Schematic of the edge coupler loss measurement setup.

uncleaved fiber, followed by a cleave to form the two fiber probes. The setup is located in a lab that has environmental control, but even small environmental fluctuations, such as temperature and humidity fluctuations, can impact the low-loss edge coupler measurements. To help compensate for these effects, a 50/50 fiber splitter at the output of the laser is used to obtain a baseline optical power reference. Due to small changes in the laser output power, the reference is constantly monitored during measurements, and the measured signal is corrected to account for the small changes. However, there can still be a drift of the reference relative to the signal due to, e.g., changes in the environment affecting the 50/50 splitter coupler and relative drift of the different photodetectors. This drift was quantified separately using multiple-day monitoring. To account for this drift, the edge coupler is measured multiple times over several days by different operators to extract a mean and standard deviation of the insertion loss of the device. For each measurement, optimization of the transmitted optical power is performed using 6-axis stages, as well as fine polarization control for final measurements. Index matching oil is used during the probing to limit the effect of Fabry-Perot reflections during active alignment.

The measured value is corrected by subtracting the system loss and accounting for the baseline drift, and then divided by two since the device under test has both an input and output edge coupler. Finally, the propagation of the short waveguide section connecting the two edge couplers from the ends of the tapers is subtracted to arrive at the fiber-to-chip loss values.

SMF-28 chip-to-fiber coupler measurements: One chip with two measured devices, each device measured 5 times in total (with two different operators measuring on two different days). Total of 10 measurements. A loss of 29 mdB was calculated for the short waveguide section connecting the two edge couplers from the ends of the tapers (4.56mm long waveguide with a propagation loss of 6.3 dB/m).

UHNA chip-to-fiber coupling measurements: One device measured 9 times in total, over two days and with three different operators. A loss of 3.6 mdB was calculated for the short waveguide section connecting the two edge couplers from the ends of the tapers (3.3mm long waveguide with a propagation loss of 1.1d B/m).

S7. ELECTRO-OPTIC SWITCHING

In figure 5d, the free-space effective Pockels coefficient of a BTO film was obtained by directing a continuous-wave laser perpendicular to the BTO films surface. The laser, at 1550 nm, is focused to a roughly $\sim$ 30 μm 1/e^2 spot size to overlap the electrode gap between two electrodes spaced 7 μm apart and with a BTO film thickness of 112nm. A bias voltage was then applied to the electrodes while the laser signal passed through the BTO film, a quarter-wave plate, a Wollaston prism, and finally detected by a balanced photodiode. The differential signal is then read out with a lock-in amplifier and interpreted to determine the birefringence of the BTO crystal, and from that the reff value of the film. Its important to note that the r_{eff} value measured here is normal to the BTO film, while a waveguide would be in-plane with the BTO film.

Figure 5f was measured at a bias voltage of 14 V on a set of four phase shifter cutback devices. Each device had straight segments whose lengths change for each. The number and orientation of non-straight segments remain the same on each device in the set, and the DC bias voltage is applied to all segments simultaneously to interpret the loss of each device with an electric field applied. To mimic realistic device operation, these phase shifter cutbacks were first poled at 40 V before they were measured optically. Device lengths of 0.4mm, 4.4mm, 9.6mm and 16.4mm were measured, with an electrode gap of 10 μm and a BTO thickness of 135nm.

Figure 5g in the main text was measured on a 2 mm-long Mach Zehnder interferometer (MZI) with a built-in path-length difference between the two arms for passive spectral interference. The BTO phase shifter had an electode gap of 10 μm and a BTO thickness of 135 nm. To pole the device, a bias voltage of 40 V was applied before the device was measured. The voltage source was swept from 40 V down to 0 V, then from 0 V to 10 V and back down to 0 V five times to determine the repeatability of the DC electro-optic response. Excluding the first pass from 10 V to 0 V to avoid nonlinear effects due to relaxation of poled domains, all following voltage sweeps result in highly repeatable DC $V_\pi L$ values with a variation of < 0.01 V-cm.

Although not designed for highspeed operation, we observed a 3 dB small-signal frequency response of > 5 GHz for bias voltages > 20 V. Plotted in Figure S17 is the small-signal electro-optic frequency response of a Mach Zehnder interferometer with an embedded 3mm long BTO phase shifter with an electrode gap of 10 μm and a BTO thickness of 135 nm, exhibiting a 3 dB frequency response of 6.9 GHz.

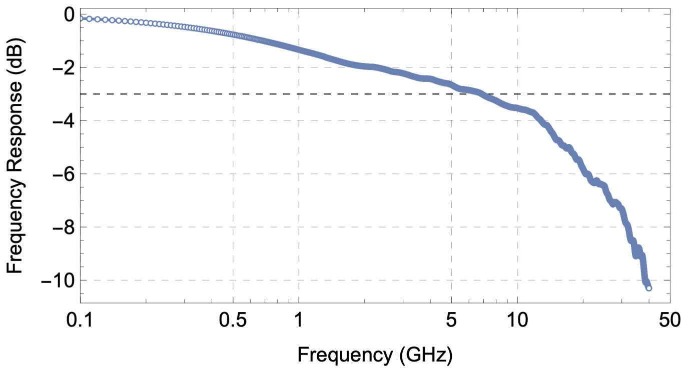

FIG. S17. Small signal frequency response for a BTO Mach Zehnder interferometer with a 6.9GHz 3dB frequency response.

S8. QUANTUM BENCHMARKING CIRCUITS

Here we provide additional details about the circuits and outline the classical systems supporting them.

S8.1. Classical Optical and Electrical Systems

In addition to the quantum optical chip and cryogenic infrastructure, the classical support infrastructure used for this demonstration can be split up into 3 major systems:

1. The Pump Distribution Network (PDN) which generates the carefully tailored pulses of pump light which drive our heralded single photon sources.
2. The Photonic Tuning Network (PTN) which is used to preform linear optic characterization of the quantum circuits, and generate the necessary electrical
3. Detector Readout Network (DRN) which amplifies and time-tags the detector clicks for analysis.

Each of these subsystems is made out of mix of commercial and custom hardware which has been integrated together mechanically into rack-mount enclosures, controlled using a linux-based custom software stack written in Python.

PDN: The PDN used for this demonstration consists of a commercial mode locked laser (Pritel UOC) operating at

1GHz repetition rate. This is pulse picked down to 125MHz using a commercial pulse picker (Pritel OPP) and amplified using a commercial erbium doped fiber amplifier (Pritel PMFA). The output of this amplifier is spectrally filtered and split to produce each optical pump output channel. Each output channel is routed through a commercial fiber optic attenuator (Oz Optics DD100), commercial fiber optic delay line (Lune ODL) and a commercial fiber optic polarization controller (Oz Optics EPC). The output of the PDN is then routed to the quantum circuit through the PTN.

PTN: The PTN used for this demonstration consists of a commercial tunable laser source (Keysight N7776C) paired

with a commercial fiber optical polarization controller (Keysight N7786C) routed to a commercial fiber optic switch (Dicon

MXR) which routes the tuning light to the quantum circuit. To detect the transmitted tuning light, commercial laser power meters (Keysight N7748C) are connected to the optical switch. Furthermore, the pump light is also routed through the optical switch in such a way that we choose to send through a tunable laser for calibration or the pump laser during quantum operation. To avoid the possibility of pump light contaminating our quantum measurements due to crosstalk in the optical switch, all of the critical optical ports of the quantum circuit (specifically, those after the pump rejection filters) are connected to a separate optical switch which only has optical power meters connected to it (no connection to the PDN). In addition to the optical components of the tuning network, the PTN drives the on-chip integrated thermal tuners which are used for tuning and control of the quantum optical circuits. We use both commercial current sources (NI PXIe-4163) and in-house designed high density current sources with 16-bit resolution.

DRN: The DRN used for this demonstration consists of commercial low noise rf amplifiers (mini-circuits ZFL-1000)

paired with a commercial bias tee (mini-circuits ZFBT-6GW) to bias the on-chip SNSPDs. The bias supply is generated using an in-house designed bias generation circuit. The amplified SNSPD clicks are routed to a commercial timetagger (Swabian) where they are tagged and sent to a computer for analysis.

S8.2. Cryogenic Assembly Thermal Requirements

Co-integration of heat-dissipating elements and superconducting detectors escalates thermal management. As a guide, at a ~ 2 Kelvin base temperatures, the available steady state cooling power is O(0.1) W/cm^2. With tuner-free robust photon sources, O(1) mW RF amplifiers, parasitic heat loads of O(0.1) mW for DC, O(1) mW for RF wires, and O(1) mW from scattered pump light per channel. We can engineer cryogenic solutions within our thermal envelope. Our switches can be used at room-temperature and do not have the same constrains. In the systems presented here, O(100) mW was successfully dissipated whilst keeping the detectors at operation temperatures.

S8.3. Integrated Heralded Single Photon Source

High-intensity pump light that imperfectly couples into the photonic circuits will scatter into the oxide cladding and silicon substrate. This presents a source of noise photons for any integrated single-photon detectors that must be mitigated. We characterize the level of scatter rejection by routing a CW source at the pump wavelength into a coupler on the device of interest that is not waveguided to any of the on-chip detectors and recording photon count rates as a function of detector bias current. The level of scatter rejection is then given by the ratio of laser power entering the chip (with any system loss calibrated out) to optical power incident on each detector at its maximum internal efficiency. We consistently measure over 115 dB of scatter rejection, see Fig. S18 for an example measurement. We also measure the source signal-to-noise ratio using a coincidence-to-accidental measurement (CAR), with an example shown in Fig. S19.

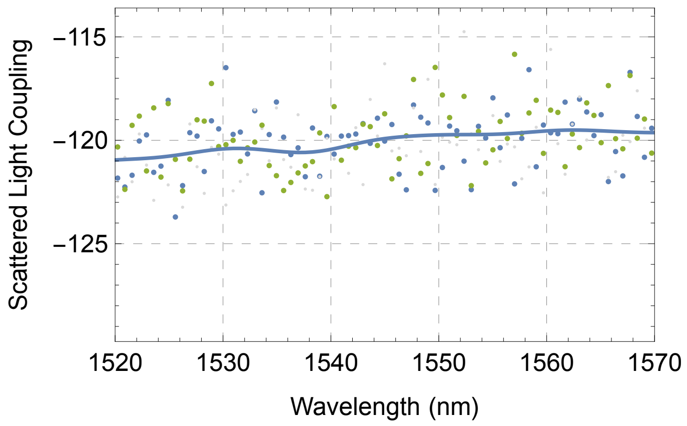

FIG. S18. Scattered light measurement with integrated single-photon detectors with scatter light mitigation structures (colours are different SNSPDs on the same PIC, solid line is a rolling-average). Input power is 1 mW, and this is the measurement light coupling as a function of wavelength, normalised to the power incident at the optical pump I/O.

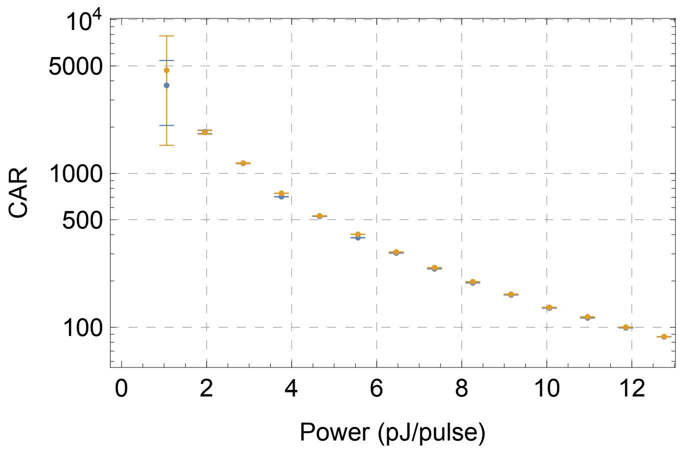

FIG. S19. Example coincidence-to-accidental measurement for two different (blue, yellow) integrated heralded photons sources on the same PIC. We vary the input power of the pump laser to vary the photon production pair rate. We observe the expected CAR $\propto 1/\text{pair rate}$ at low pump power. The CAR is calculated via CAR $= P(h,s)/(P(h)P(s))$, where $P(h)$, $P(s)\&P(h,s)$ are the probability of observing a herald, signals, and herald-signal coincidence probability respectively. A CAR > 2 signifies non-classical correlations. Error-bars computed via Poissonian counting statistics. At the maximum CAR the pair-production probability is $\sim 0.03\%$

S8.4. State Preparation and Measurement Fidelity

To estimate the average quantum state preparation and measurement (SPAM) fidelity we first prepare a qubit, and then immediately measure the qubit. Our circuit prepares spatially encoded qubits via a series of balanced tunablable MZIs with reconfigurable internal phases.

We prepare many independent and identical copies of all six single qubit Pauli eigenstates and measure them in the three Pauli bases $\sigma_x, \sigma_y, \sigma_z$. Quantum state estimation is used to reconstruct the maximum likelihood state for each of the prepared eigenstates. We use the Uhlmann Fidelity as a measure of the quantum distance between the measured state and target state. To obtain the uncertainty on the reconstructed fidelity, we compute the Bayesian posterior probability distribution on each state, utilizing a uniform prior distribution according to the Bures metric. We use the posterior distribution to sample many states and compute their fidelities and report their mean and standard deviation. Mean state fidelity across the prepared input states in the Pauli set was $(99.98 \pm 0.01)\%$.

We separate SPAM errors due to imperfect single-photon detection (loss, noise clicks) from errors due to imperfect gates by sending CW coherent light through the single qubit benchmarking circuit (Figure 3a in the main text) and performing tomography using off-chip photo-diodes. State fidelity produced from this measurement is limited by phase miscalibrations and imperfections in the passive photonic structures. When we probed this way, the mean state fidelity across the prepared input states in the Pauli set was $(99.996 \pm 0.003)\%$ with individual state fidelities exceeding 99.999%..

S8.5. Chip-to-chip qubit interconnect

Photonic modules are connected via optical fibre in photonic quantum computers. Two common ways to transfer light from on-chip single-mode waveugides to optical fibres is either via low-loss edge couplers, or vertical grating couplers. In this chip-to-chip qubit demonstration we utilize vertical grating couplers, implementing two-dimension grating couplers to simultaneous couple horizontal and vertical polarizations, similar to the work presented in [18]. We design, build and measure 2D grating couplers optimized at a 15-degree angle of incidence, optimizing the design at the same angle as our commensurate 1D grating couplers. The design incorporates a uniform circular lattice of 11 μm x 11 μm pitch area. We optimize the diameter and pitch of the lattice structures to simultaneously maximize the coupling and polarization-path extinction ratio of the device.

To validate the qubit transfer, we prepare photonic heralded single qubits on a transmitter device, and transport them to a receiver chip via SMF-28 optical fiber. The dual-rail and polarization qubit encodings are transferred twice via a two-dimensional grating coupler on each circuit. We utilize quantum process tomography of the path encoded states measured by integrated SNSPD's on-chip at the receiver to benchmark and bound the total channel fidelity. We measure a post-selected mean state fidelity of $99.81\% \pm 0.08\%$.

One main drawback of the two-dimension grating coupler, however, is the typically high insertion loss of these devices making them impractical for efficient transport of single photons. This demonstration acts as a proof-of-principle, where future platforms can simultaneously show high fidelity and low loss by using on-chip polarization splitters and low-loss edge couplers.

S8.6. HOM interference

Hong-Ou-Mandel interference is measured by photons generated within independent AMZI-coupled sources where both the bus and resonator phases can be independently tuned with thermal phaseshifters. To optimize the tuning, we record the spectrum of the source for an array of values and perform a joint optimization over the currents to align the central resonance wavelength and minimize the source linewidth.

Once tuned, we characterize each source with a range of photon counting measurements as a function of the pump power. To generate single photons, we utilize the supporting systems listed above (PDN, PTN, DRN). The actively mode-locked laser with a Gaussian spectrum of 300pm full width half maximum (FWHM) and subsequently filter the spectrum using a fiber-based dense wavelength division multiplexing unit (DWDM) with a 100 GHz bandwidth. The 125 MHz repetition rate pulses are delivered via a packaged fiber-array unit coupling to on-device grating couplers. Off-chip programmable variable optical attenuators adjust the pulse energy for each source independently. We record photon countrates and bi-directional signal-herald histograms across all detector combinations. The coincidence-to-accidentals ratio (CAR) and the signal and herald Klyshko efficiencies are calculated as a function of the attenuation (Fig.S20).

The HOM circuit integrates a tunable beam splitter formed by an on-device MZI. We characterize the MZI to extract the tuning current that optimizes a 50:50 splitting ratio. In this setting, 1, 2 and 3-fold photon measurements are recorded to determine the heralded g2. The pump power is set to deliver photons with a CAR of 100 (high power setting) and 900 (low power setting), with four-fold click rates at minimum of HOM dip of $\sim$ 1 Hz and $\sim$ 1 mHZ respectively (Fig.S20).

The delay between single photons in the HOM circuit is adjusted off chip via programmable, motorized delays on each pump laser within the PDN. HOM interference at high power is collected across a range of delays to determine the optimal delay setting which maximizes interference visibility. The HOM interference is then collected at low power around the minimum delay. We also confirm we have only a small degradation ($\sim$ 0.05%) in HOM visibility by residual pump noise, by matching our measurements to our model (Fig.S21).

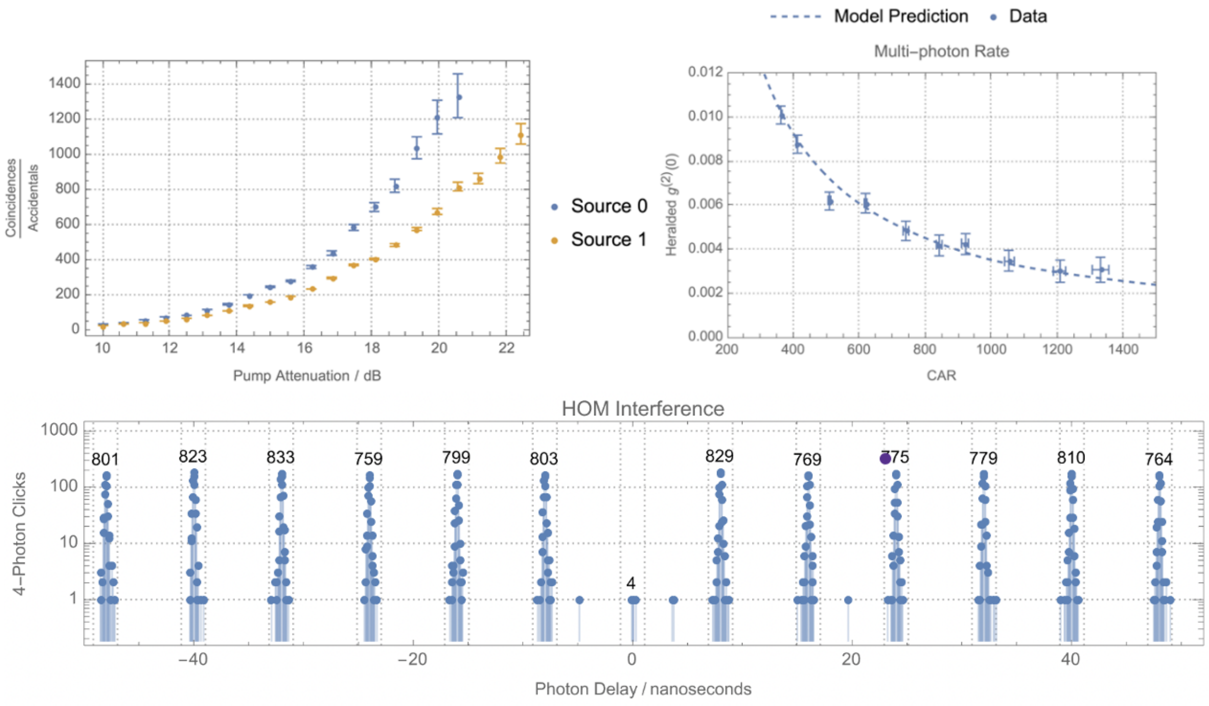

FIG. S20. (Top left) Measured CAR as a function of laser attenuation. (Top, right) Heralded single photon $g^{(2)}(0)$ measurements as a function of source coincidences-to-accidentals rate (blue data points) and predicted trend (dashed line). (Bottom) Measured four-photon coincidence events histogram as a function of time delay between photon arrival. Each peak is integrated over a 2ns window centred around the expected value. The integration window is shown by dashed vertical grid-lines. The total number of counts per integration is written above each dataset. HOM interference occurs when the photon delay is zero.

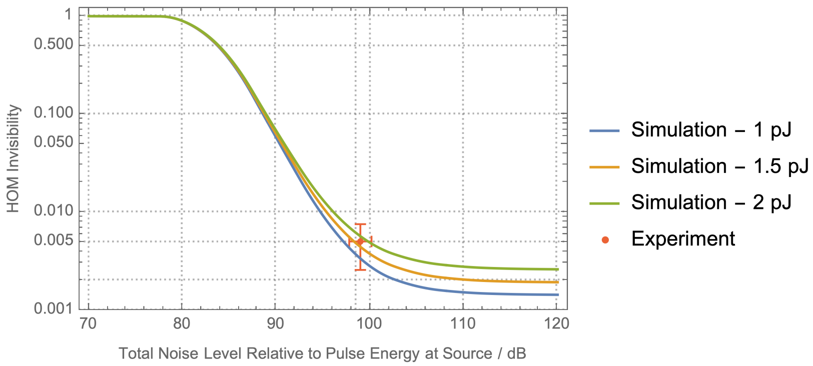

FIG. S21. Simulated dependence on HOM error rate (where HOM invisibility = 1 - visibility) as a function of total noise level at detection. Noise level is defined as total (non-source origin) photon detection event probability, normalized relative to the average number of photons per pulse.

S8.7. Two-qubit fusion

To demonstrate the fusion of two dual-rail qubits, we prepare a entangled two qubit state, $|\Psi^-\rangle$, by injecting $|+-\rangle$, states into a configurable path-encoded fusion network. To create the entangled state, a pair of heralded photons are injected into a reconfigurable two qubit circuit, with initial state

$$|\psi\rangle_0 = \hat{a}^\dagger_{0,0}\hat{a}^\dagger_{1,1}\,|\text{vac}\rangle\,, \tag{23}$$

where $\hat{a}^\dagger_{a,x}$ represents the single photon creation operator for qubit $a \in \{0,1\}$ in waveguide $x \in \{0,1\}$. The single photons are initially transformed via a pair of path encoded beam splitters formed by two directional couplers that apply local hadamards to each qubit such that the on-chip state becomes

$$|\psi\rangle_0 \rightarrow \frac{1}{2}(\hat{a}^\dagger_{0,0}+\hat{a}^\dagger_{0,1})(\hat{a}^\dagger_{1,0}-\hat{a}^\dagger_{1,1})\,|\text{vac}\rangle\,. \tag{24}$$

To complete the fusion we apply a qubit parity-check operation, where odd parity states are transformed onto the coincidence basis (qubit subspace) $\{\,|1010\rangle,\,|1001\rangle,\,|0110\rangle,\,|0101\rangle\,\}$ (following Fock notation, across the modes, (a,x), $(0,0),(0,1),(1,1),(1,0)$ respectively), and even parity states are transformed out of the coincidence basis onto $\{\,|1100\rangle,\,|0011\rangle\,\}$. On device, this is achieved via a tunable MZI that is thermally tuned to swap the waveguide modes such that $\hat{a}^\dagger_{0,1} \leftrightarrow \hat{a}^\dagger_{1,0}$ where the state becomes

$$\begin{aligned}|\psi\rangle_0 &\rightarrow \frac{1}{2}(\hat{a}^\dagger_{0,0}+\hat{a}^\dagger_{1,0})(\hat{a}^\dagger_{0,1}-\hat{a}^\dagger_{1,1})\,|\text{vac}\rangle\\ &= \frac{1}{2}\big(\underbrace{\hat{a}^\dagger_{1,0}\hat{a}^\dagger_{0,1}-\hat{a}^\dagger_{0,0}\hat{a}^\dagger_{1,1}}_{\text{Qubit Subspace}}+\hat{a}^\dagger_{0,0}\hat{a}^\dagger_{0,1}-\hat{a}^\dagger_{1,0}\hat{a}^\dagger_{1,1}\big)\,|\text{vac}\rangle\end{aligned} \tag{25}$$

where the projection onto the $|\Psi^-\rangle$ state is post-selected on successful coincidence measurements with 50% probability.

To validate the output, we directly measure the Uhlmann Fidelity by decomposing the target state into locally measurable observables in the local Pauli basis and subsequently measure their expectation values. In this method, one can group the decomposed target density matrix into two locally observable terms referred to as the Population, $\hat{P}$, and Coherence, $\hat{C}$, in the following way

$$\begin{aligned}\rho_{\Psi^-} &= \Psi^-\Psi^-\\ &= \frac{1}{2}\big(\underbrace{\hat{I}\otimes\hat{I}-\hat{Z}\otimes\hat{Z}}_{\text{Population, }\hat{P}}\big)+\frac{1}{2}\big(\underbrace{-\hat{X}\otimes\hat{X}-\hat{Y}\otimes\hat{Y}}_{\text{Coherence, }\hat{C}}\big),\end{aligned} \tag{26}$$

where $\hat{I},\hat{Z},\hat{X},\hat{Y}$ represent the usual four Pauli matrices.

Here the Population term measures the degree of correlation of the qubits, given by the target states diagonal density matrix elements and limited by the classical single photon interference visibility. The Coherence term measures the degree of quantum

superposition of the state and is limited by the quantum interference visibility. To compute the state fidelity, F, we measure the mean of the expectation values of $\langle \hat{P} \rangle$ and $\langle \hat{C} \rangle$.

$$\begin{aligned} F_{\text{Uhlmann}} &= \left(\sqrt{\sqrt{\rho_{\Psi^-}}\sigma\sqrt{\rho_{\Psi^-}}} \right)^2 \\ &= \left(\rho_{\Psi^-}\sigma \right) \\ &= \frac{\left(\hat{P}\sigma \right) + \left(\hat{C}\sigma \right)}{2} \\ &= \frac{\langle \hat{P} \rangle + \langle \hat{C} \rangle}{2} \end{aligned} \tag{27}$$

To measure the expectation values, the prepared states are evolved via a pair of arbitrary local qubit projections capable of full state tomography. The state projectors are configured to individually measure the eigenstates of each operator.

In our experiment, we first measure the output state for where the qubits are comprised of fully distinguishable photons. In this case, we expect to measure a mixed state that interferes classically but has zero quantum coherence. In this instance, we measure the Population fidelity 0.9980 ± 0.0004 and Coherence fidelity as 0.003 ± 0.004 leading to a Fidelity of 0.5003 ± 0.0023, consistent with a classically correlated state which has a maximum fidelity of 0.5. We then repeat the measurement with pure & indistinguishable photons. In this case, a population of 0.9975 ± 0.0011 and coherence of 0.9869 ± 0.0022 is measured (see photon arrival histogram in Fig.S22), leading to a state fidelity of 0.9922 ± 0.0012. Using quantum state estimation, we compute the maximum likelihood state for both the distinguishable and identical photon cases. The density matrices of these results are shown in Figure 3 in the main text.

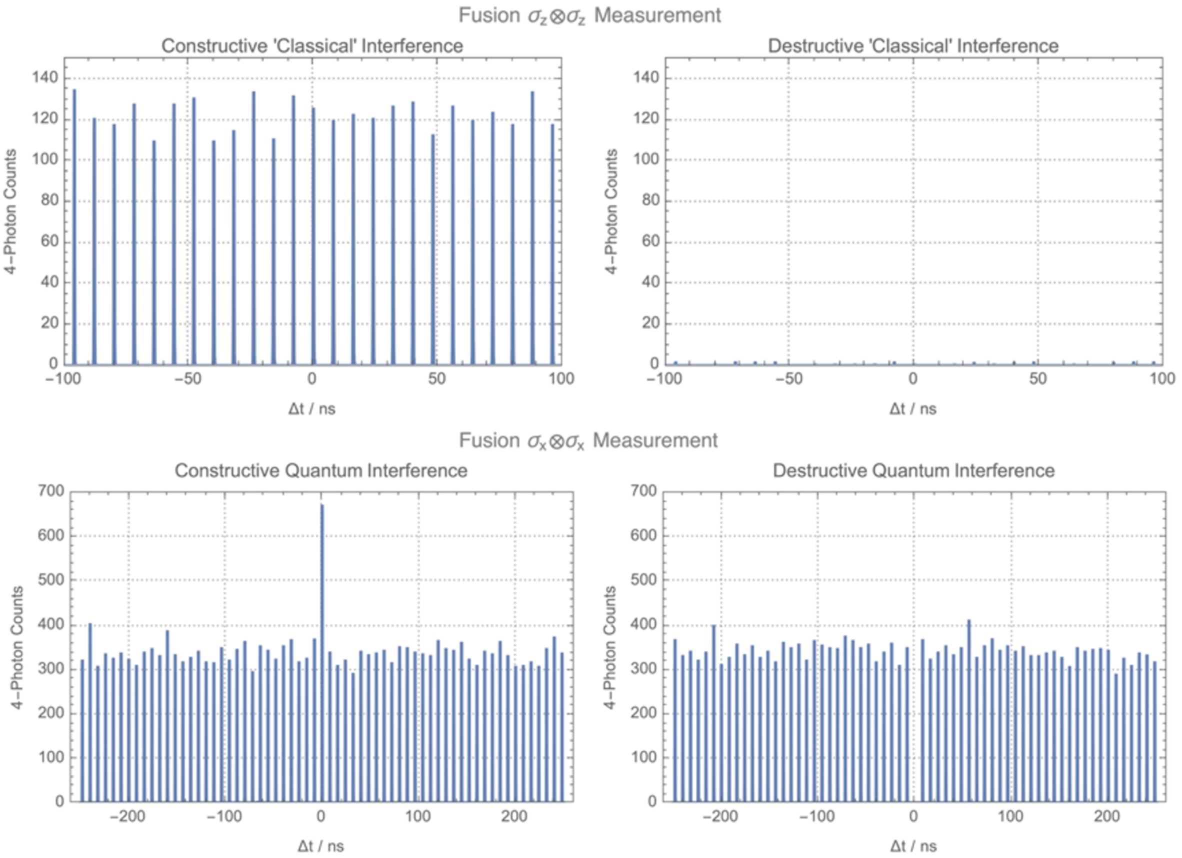

FIG. S22. Heralded two-photon coincidence event histograms. Heralded photon events are counted as a function of relative photon arrival time, where at $\Delta t = 0$ represents four-fold coincidence events that arise from the same laser pulse. (top) Histogram events where the projective measurements are set to the computational basis. (Bottom) Histogram events where the projective measurements are set to the $\hat{X} \otimes \hat{X}$ basis. In each case, the left plot shows the constructive (negative) eigenstates and right shows the destructive (positive) eigenstates. The resolution of each histogram is 50 picoseconds where the total coincidence events are integrated using a 1 nanosecond window.

[1] E. Knill, R. Laflamme, and G. J. Milburn, Nature **409**, 46 (2001).
[2] T. C. Ralph, A. G. White, W. J. Munro, and G. J. Milburn, Phys. Rev. A **65**, 012314 (2001).
[3] N. Yoran and B. Reznik, Phys. Rev. Lett. **91**, 037903 (2003).
[4] M. A. Nielsen, Phys. Rev. Lett. **93**, 040503 (2004).
[5] D. E. Browne and T. Rudolph, Phys. Rev. Lett. **95**, 010501 (2005).
[6] C. M. Dawson, H. L. Haselgrove, and M. A. Nielsen, Phys. Rev. Lett. **96**, 020501 (2006).
[7] R. Raussendorf and H. J. Briegel, Phys. Rev. Lett. **86**, 5188 (2001).
[8] S. Bartolucci *et al.*, Nature Communications **14**, 912 (2023).
[9] A. L. Migdall, D. Branning, and S. Castelletto, Phys. Rev. A **66**, 053805 (2002).
[10] T. B. Pittman, B. C. Jacobs, and J. D. Franson, Phys. Rev. A **66**, 042303 (2002).
[11] E. Meyer-Scott, C. Silberhorn, and A. Migdall, Review of Scientific Instruments **91**, 041101 (2020).
[12] S. Bartolucci, *et al.*, (2021), arXiv:2109.13760 [quant-ph].
[13] H. Bombin *et al.*, (2021), arXiv:2103.08612 [quant-ph].
[14] W. P. Grice, A. B. U'Ren, and I. A. Walmsley, Phys. Rev. A **64**, 063815 (2001).
[15] M. Avenhaus, A. Eckstein, P. J. Mosley, and C. Silberhorn, Opt. Lett. **34**, 2873 (2009).
[16] P. Mosley, *Generation of heralded single photons in pure quantum states*, Ph.D. thesis, University of Oxford (2007).
[17] A. Christ, K. Laiho, A. Eckstein, K. N. Cassemiro, and C. Silberhorn, New Journal of Physics **13**, 033027 (2011).
[18] D. Llewellyn *et al.*, Nature Physics **16**, 148 (2020).
